# Factors Influencing the Implementation and Adoption of Digital Nursing Technologies: Systematic Umbrella Review

**DOI:** 10.2196/64616

**Published:** 2025-07-31

**Authors:** Stefan Walzer, Christoph Armbruster, Sonja Mahler, Erik Farin-Glattacker, Christophe Kunze

**Affiliations:** 1 Care & Technology Lab, Furtwangen University Furtwangen Germany; 2 Section of Health Care Research and Rehabilitation Research, Institute of Medical Biometry and Statistics Medical Faculty and Medical Center - University of Freiburg Freiburg Germany

**Keywords:** digital, technology, care, nursing, implementation, adoption, umbrella review

## Abstract

**Background:**

Digital nursing technologies (DNTs) are a promising solution to address challenges in health care systems, such as demographic shifts, nursing shortages, or difficulties in retaining nurses. Despite their potential benefits, the integration of DNTs into care settings remains complex due to multiple factors influencing their implementation and adoption.

**Objective:**

We aimed to examine factors that influence the implementation and adoption of DNTs used in nursing care settings from the perspective of nurses.

**Methods:**

We used an umbrella review methodology to synthesize the evidence on DNTs and the complexities of their implementation. We searched for systematic reviews that focused on DNTs in formal care settings across 4 databases (PubMed, CINAHL, Cochrane Library, and Business Source Premier) and examined reference lists of the included reviews published in English until January 2025. Two researchers independently performed data extraction and quality assessment. Data analysis was structured by embedding the results in the NASSS (nonadoption, abandonment, scale-up, spread, and sustainability) framework, a model for explaining the adoption and abandonment of health and care technologies, as well as challenges to their scaling, diffusion, and sustainability. Reporting of this study adhered to the PRISMA (Preferred Reporting Items for Systematic Reviews and Meta-Analyses) checklist.

**Results:**

A total of 4803 reviews were identified, of which 65 (1.36%) met the inclusion criteria. We identified 52 influencing factors across 6 NASSS domains, with particular emphasis on adopter-related barriers and facilitators. Key barriers included insufficient training, increased workload, and low technological confidence, which impacted efficiency and the quality of care. In addition, concerns regarding professional role, autonomy, and privacy influenced nurses’ acceptance of DNTs. Facilitators included leadership support, a positive corporate culture, and targeted training initiatives.

**Conclusions:**

We synthesized key facilitators and barriers to implementation and adoption of DNTs in nursing care. Leadership support, adequate training, and alignment with care needs drive successful implementation, while resource constraints and workflow disruptions pose challenges. Addressing both technological requirements and nursing needs is critical. Future research should focus on long-term studies and practical tools to support stakeholders in effectively integrating DNTs into nursing practice.

**Trial Registration:**

OSF Registries 10.17605/OSF.IO/BG8CY; https://osf.io/bg8cy

## Introduction

### Background

In the context of pressing challenges, such as workforce shortages and aging populations [1], digital technologies have emerged as a potential means to improve the working conditions of health care professionals while enhancing the quality of care [2,3]. As a result, research on digital health technologies has expanded significantly across a range of disciplines, including engineering, medicine, nursing, psychology, philosophy, and sociology [4]. However, the interdisciplinary nature and broad scope of this research landscape make it increasingly complex and difficult to navigate [5].

Despite the growing interest in this area, most studies and reviews of digital health technologies tend to focus primarily on physician or patient outcomes, although nurses constitute approximately 44.7% of the global health workforce, accounting for around 27.9 million nurses worldwide [1]. Given their central role in patient care, nurses are key users of digital nursing technologies (DNTs) and play a crucial role in their adoption and effective implementation in nursing care settings [6]. DNTs are tools designed to help nurses provide high-quality care by facilitating rapid decision-making (eg, decision support systems for adherence to clinical guidelines) or complementing nursing tasks with technological solutions (eg, robots that assess vital signs before consultations or sensors that detect bed-exit events to prevent falls) [7]. These technologies serve multiple purposes, including enhancing patient safety, improving workflow efficiency, and reducing the physical and cognitive burden on nurses [8]. DNTs can function as stand-alone tools for individual nurses or be embedded in broader organizational care processes, such as integrated electronic health record systems or automated medication dispensing systems. By addressing specific clinical or operational needs, DNTs aim to optimize resource use and support nurses in managing complex care environments [7,8]. A detailed document outlining the DNT categories and definitions is provided in Multimedia Appendix 1 [7].

In recent years, there has been an increasing number of research and innovation initiatives at national and international levels, driven by high expectations of improving care through the implementation of DNTs [8-10]. However, the rate of successful implementation and sustainable uptake remains rather low, regardless of these efforts [7,11]. This may be due to a range of factors, such as organizational resistance [9], limited infrastructure [10], or insufficient training [11], which hinder the seamless integration of DNTs into care workflows and limit their potential to transform nursing practice. The complex and dynamic nature of the nursing care setting makes it challenging to determine clear cause-and-effect relationships in technology implementation [12]. DNTs can have both positive and negative impacts, which may not always be immediately apparent or easily measured. For example, a study of the implementation of a new patient monitoring system [13] found that the system’s real-time alerts reduced response times to critical situations but also may have led to alarm fatigue among nurses, ultimately reducing their effectiveness in responding to urgent alerts. In addition, changes in health care policy or budget constraints can affect the availability of necessary infrastructure or training programs, further complicating the process of understanding the factors that lead to the successful implementation and adoption, or the failure, of DNTs [14].

Although several evaluation frameworks for digital technologies exist [14-18], as well as scoping and systematic reviews that examine different types (eg, monitoring, sensors, and robots); settings (eg, formal and informal care); and outcomes (eg, acceptability, effectiveness, and efficiency) [7,8,19], significant gaps remain in understanding the factors that influence the implementation and adoption of DNTs.

### Objectives

We aimed to explore the factors (barriers and facilitators) that influence the implementation and adoption of DNTs used in nursing care settings from the perspective of nurses.

Our paper contributes by synthesizing existing evidence through an umbrella review approach, providing a comprehensive overview of the key factors influencing DNT implementation and adoption. To our knowledge, no existing review aims to synthesize and map all relevant influencing factors across the spectrum of implementation and adoption for DNTs in the context of formal care.

By identifying these key factors, stakeholders can better address barriers, tailor interventions, and create environments that foster the effective integration of DNTs.

## Methods

### Design

The PRISMA (Preferred Reporting Items for Systematic Reviews and Meta-Analyses) checklist was used to conduct and report our findings [20].

Due to the large number of relevant reviews, we decided to conduct an umbrella review to compile, synthesize, and summarize the results of several systematic reviews [21]. This design allows us to provide a comprehensive overview of the current landscape of DNTs to support nursing care across settings and interventions [21]. In addition, we aimed to identify potential gaps in the existing evidence to guide the direction of future research initiatives. The umbrella review framework is conceptualized as a systematic review in which only reviews are used as the fundamental unit of analysis within the overarching structure [22]. This methodological approach is particularly well suited to broad areas of inquiry, where the aim is to provide a consolidated summary of the most rigorously conducted reviews of advances within a particular field [22]. Using this approach, it is possible to identify gaps in the existing literature and to synthesize and present the most important findings [22]. The review protocol was registered in the Open Science Framework [23]. Minor amendments were made to the study protocol during the peer-review process. These included a revision of the study title and an extension of the literature search period.

### Search Strategy

A sensitive search was conducted using the PICO (population, intervention, comparison, outcome) scheme to guide the systematic review process [24].

We performed a systematic literature search in 4 databases (PubMed, CINAHL, Cochrane Library, and Business Source Premier) between March 2023 and May 2023. Although no specific number of databases is required for an umbrella review, the selection should be based on the research question and the scope of the review [25]. The four databases used in this review were chosen to ensure coverage of literature relevant to our topic due to their strong thematic relevance. During the preliminary phase, we conducted a pilot search to refine the search strategy (SW and CA). Keywords (including MeSH [Medical Subject Headings] terms and synonyms) were grouped into three overarching categories: (1) DNT, (2) implementation, and (3) formal care. These categories were linked using the logical operator “AND,” while terms within each category were linked using “OR.” The search terms were tailored to the specific criteria of each database.

The results of the pilot search were reviewed for appropriateness, and the search strategy for the main search was iteratively refined. To ensure methodological rigor, the final search strategy was validated in collaboration with a library consultant from the Furtwangen University. The search was updated in December 2024 and January 2025 to include the most recent publications. We also screened the reference lists of the included reviews to reduce the risk of missing relevant articles. The complete search strategy, along with database-specific adjustments, are provided in Multimedia Appendix 2.

### Inclusion and Exclusion Criteria

The inclusion and exclusion criteria were defined based on the PICO framework [24], focusing on the population (nurses), the intervention (DNTs), and the outcome (factors influencing the implementation and adoption of DNTs). The comparison component was not applicable to this study and was therefore excluded. Refer to Textbox 1 for details. Studies had to meet the following inclusion criteria: reviews where nurses were either the primary focus or where findings on nurses’ perspectives could be meaningfully extracted were included; reviews including mixed professional groups (eg, physicians, therapists, and nurses) were only included if they reported clearly distinguishable results relevant to nurses; only knowledge synthesis publications, such as frameworks and reviews, were considered; and studies written in English or German and published between 2006 and 2024 were included.

**Inclusion and exclusion criteria.**

**Inclusion criteria**

- Population: nurses and other health care professionals, including nurses
- Intervention: digital nursing technologies (DNTs), categorized according to Multimedia Appendix 1. Additionally, technologies not only used by nurses (eg, electronic medical records [EMRs]), but also clearly relevant to nursing practice, are included.
- Outcome: factors influencing the introduction, implementation, or adoption, and target criteria related to the influencing factors (eg, attitude and feasibility)
- Study type: synthesis of studies (systematic reviews) based on qualitative or quantitative primary studies, peer-reviewed articles, and open-access articles.
- Language: English and German

**Exclusion criteria**

- Population: only other health professionals (eg, physicians), relatives only, and patients only
- Intervention: only other technologies, only other objectives or end points, and purely technical evaluations of technologies
- Outcome: studies that do not address influencing factors
- Study type: empirical studies (qualitative and quantitative primary studies, mixed methods designs, and reviews of empirical studies) and studies that do not focus on the results of other studies
- Language: other languages

The exclusion criteria were applied to studies that did not contain results regarding factors influencing the implementation and adoption of DNTs in the context of nursing care. Primary studies, randomized controlled trials, books, dissertations, and gray literature were also excluded, as these types of publications do not meet the level of knowledge synthesis required for this type of review [21]. In addition, a temporal exclusion criterion was enforced to exclude studies published before 2006. This was done because such articles may not provide relevant evidence, as the technologies are likely to be outdated by that time [26].

### Data Extraction and Quality Assessment

During the initial screening phase, which included title and abstract screening, each article was individually assessed by at least 2 reviewers (SW and CA). Disagreements were resolved through constructive dialogue and when necessary, by a third reviewer (CK). The subsequent full-text review followed the same protocol. The articles that met all the inclusion criteria were thoroughly reviewed using a structured data extraction form.

The data extraction form was used to extract relevant details covering study attributes (eg, title, year, authorship, country, review type, and the number of studies included) and details of integrated technologies and any content relevant to implementation aspects, including identifiable barriers and facilitators. Data extraction was performed independently by 2 reviewers (SW and SM). The resulting data extraction was reviewed and refined for each article.

To assess the quality of the included studies, we used the Joanna Briggs Institute Critical Appraisal Checklist for Systematic Reviews and Research Syntheses [27]. This checklist is a widely accepted and validated tool designed to ensure the methodological rigor and transparency of systematic reviews. It includes 10 criteria that address key aspects of the review process, including the clarity of the review question, the appropriateness of the inclusion and exclusion criteria, the transparency of the study selection process, and the comprehensiveness of the search strategy. Other important dimensions assessed include the standardization of the data extraction process, the thoroughness of the quality assessment of the included studies, the appropriateness of the synthesis methods, the consistency of the conclusions with the findings, and the identification and reporting of potential conflicts of interest.

Each included review was evaluated against these criteria and a score was assigned to reflect its overall methodological quality. The scoring process resulted in an overall score ranging from 0% to 100%, providing a quantitative measure of quality. Higher scores indicated reviews with robust and transparent methodologies, while lower scores highlighted areas of potential methodological weakness. The quality assessment was carried out independently by 2 researchers (SW and SM) to ensure an objective and systematic assessment. Any disagreements in the assessment were resolved by discussion to reach consensus. Multimedia Appendix 3 [9,10,19,27-89] provides a detailed scoring document.

### Data Synthesis and Analysis

**Figure 1:**
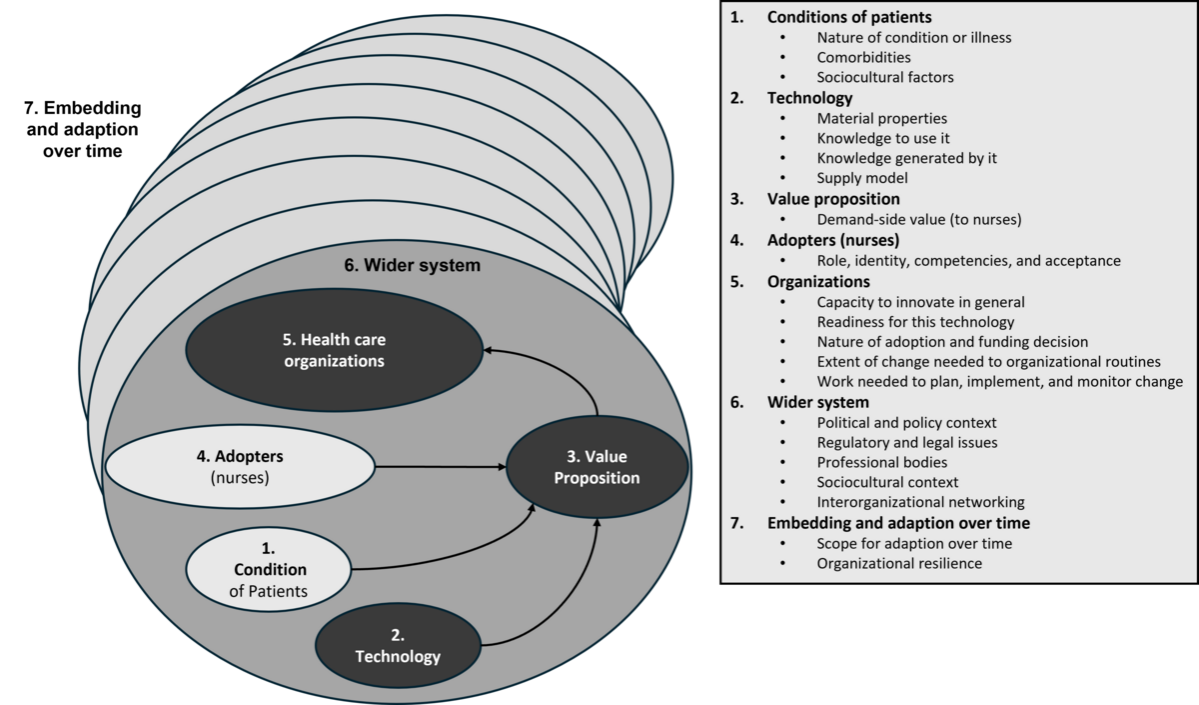
The NASSS (nonadoption, abandonment, scale-up, spread, and sustainability) framework (adapted from Greenhalgh et al [14]).

We applied the NASSS (nonadoption, abandonment, scale-up, spread, and sustainability) framework as a methodological tool to categorize the data (Figure 1) [14]. The NASSS framework was selected due to its ability to capture the multifaceted nature of technology implementation in health and care settings. It considers multiple levels of analysis to predict and evaluate the success of technology implementation and adoption. Failures, partial successes, and unexpected problems with technology are explained by examining complex interactions across 7 domains: condition, technology, value proposition, adopter system, organization, broader context, and changes over time [14].

The coding scheme was developed iteratively and refined by consensus among 3 researchers (SW, CA, and SM). Following the study by Kuckartz and Rädiker [90], we deductively categorized relevant data fragments into 1 of the 7 domains of the NASSS framework. The selected fragments were then inductively categorized into subthemes. Rather than analyzing each technology or care setting separately, we integrated the identified factors into overarching categories to capture broader implementation barriers and facilitators. This approach allowed us to synthesize common themes across diverse technologies and settings while still acknowledging contextual nuances.

By structuring the findings in this way, we aimed to highlight general barriers and facilitators rather than treating each setting or technology in isolation. We systematically mapped each code to the corresponding domain, ensuring a structured categorization of influencing factors. For example, factors categorized under the “technology” domain included usability and integration issues as barriers, whereas intuitive design and interoperability were identified as facilitators. Similarly, the “adopters” domain captured aspects such as technological confidence and workload, highlighting both enabling and inhibiting influences on DNT implementation.

## Results

### Characteristics of the Included Studies

**Figure 2:**
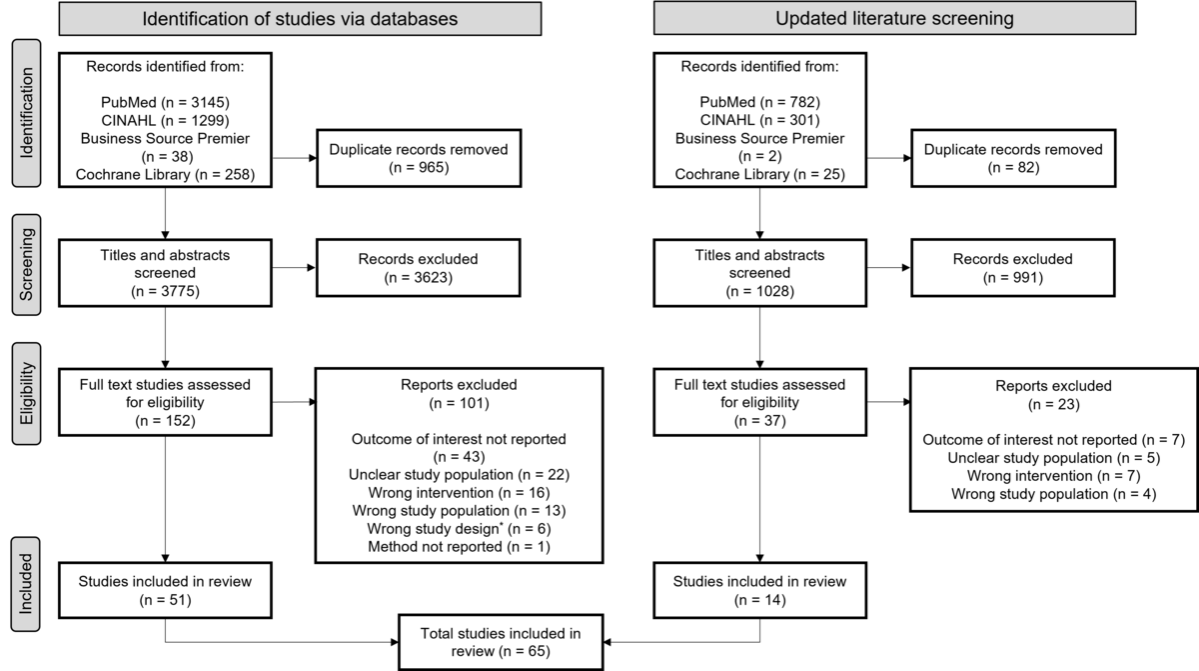
PRISMA flow diagram of the study selection process. *Six articles were not systematic reviews, which only became apparent in the full-text screening.

Figure 2 illustrates the selection and screening process, including the primary reasons for exclusion. The initial search identified 4740 articles, from which 965 (20.36%) duplicates were removed. A total of 79.64% (3775/4740) of the articles underwent abstract screening, resulting in 4.03% (152/3775) of full-text assessments. Of these 152 articles, 101 (66.4%) studies were excluded due to various reasons (eg, outcome of interest not reported and wrong study design).

An updated literature screening identified an additional 1110 records, with 82 (7.39%) duplicates removed, leaving 1028 (92.61%) titles and abstracts for screening. After excluding 96.4% (991/1028) of the articles, 3.6% (37/1028) of the full texts were assessed, of which 62% (23/37) of the studies were excluded. Ultimately, 38% (14/37) of the additional articles were included, leading to a total of 65 studies in this review. Across these 65 reviews, a total of 1806 individual studies were synthesized. The number of studies included per review ranged from 5 to 140, with a median of 18.5 studies per review.

A total of 65 studies on DNT published between 2010 and 2024 were analyzed, with 46 (71%) studies published after 2018. The studies were conducted in 24 countries, with most originating from the United States (15/65, 23%), followed by Australia (7/65, 11%), Finland (6/65, 9%), and Germany (4/65, 6%). With regard to study types, systematic reviews dominated (26/65, 40%), followed by integrative reviews (13/65, 20%); scoping reviews (10/65, 15%); and a smaller number (16/65, 25%) of literature, narrative, rapid and mixed methods reviews. The average quality rating was 92% (SD 11.3%), with 65% (42/65) of the studies scoring 100%.

The studies focused on interventions that included technologies from different technology categories (11/65, 17%), decision support systems (10/65, 15%), hospital or care institution information systems (10/65, 15%), telehealth (9/65, 14%), and electronic medical record systems (7/65, 11%). Reviews focusing on clinical decision support systems (10/65, 15%; 376 included studies; mean 37.6 studies per review), hospital information systems (10/65, 15%; 295 studies; mean 29.5 studies per review) and telehealth (9/65, 14%; 227 studies; mean 25.2 studies per review) tended to include a higher number of primary studies. In contrast, assistive devices (5/65, 8%; 69 studies; mean 13.8 studies per review), and tracking technologies (1/65, 2%; 8 studies; mean 8.0 studies per review) included markedly fewer studies. Multimedia Appendix 1 provides more details on categories and definitions of DNTs.

There was variability in the use of implementation-related terminology and the focus of the included studies. In addition, the study settings varied, including inpatient and outpatient settings, such as home visiting programs, intensive care units, and nursing homes. Refer to Table 1 for more details on the characteristics of the included studies.

**Table 1:** Characteristics of the included studies.

Study and year	Title	Country	Type of review	Included studies, n	Quality appraisal (%)	Type of digital nursing technology
Waneka and Spetz [28], 2010	Hospital information technology systems’ impact on nurses and nursing care	United States	Systematic review	74	72%	Multiple technologies
Stevenson et al [29], 2010	Nurses’ experience of using electronic patient records in everyday practice in acute/inpatient ward settings: a literature review	Sweden	Literature review	5	93%	Electronic medical records
Wulff et al [30], 2011	Medication administration technologies and patient safety: a mixed-method systematic review	Canada	Systematic review	12	89%	Assistive device
Young et al [31], 2011	Staff acceptance of Tele-ICU coverage: a systematic review	United States	Systematic review	23	67%	telehealth
San et al [32], 2012	Factors affecting registered nurses’ use of medication administration technology in acute care settings: a systematic review	Singapore	Systematic review	6	100%	Assistive device
Kosse et al [33], 2013	Sensor technologies aiming at fall prevention in institutionalized old adults: a synthesis of current knowledge	Netherlands	Systematic review	12	100%	Sensor technologies
Kumar et al [34], 2013	Tele-ICU: efficacy and cost-effectiveness approach of remotely managing the critical care	United States	Literature review	25	86%	Telehealth
Piscotty and Kalisch [35], 2014	Nurses’ use of clinical decision support: a literature review	United States	Literature review	20	86%	Decision support
Zhang et al [36], 2014	Nurses’ attitudes towards medical devices in healthcare delivery: a systematic review	China	Systematic review	30	100%	Multiple technologies
Teh et al [37], 2015	Clinical effectiveness of and attitudes and beliefs of health professionals towards the use of health technology in falls prevention among older adults	Australia	Systematic review	17	89%	Multiple technologies
Strudwick [38], 2015	Predicting nurses’ use of healthcare technology using the technology acceptance model: an integrative review	Canada	Integrative review	20	100%	Multiple technologies
Radhakrishnan et al [39], 2016	Barriers and facilitators for sustainability of tele-homecare programs: a systematic review	United States	Systematic review	16	89%	Telehealth
Penny et al [40], 2018	Registered nurse and midwife experiences of using videoconferencing in practice: a systematic review of qualitative studies	Australia	Systematic review	9	100%	Telehealth
Mileski et al [41], 2017	Adopting telemedicine for the self-management of hypertension: systematic review	United States	Systematic review	14	56%	Telehealth
Kaye [42], 2017	Nurses’ attitudes toward meaningful use technologies: an integrative review	United States	Integrative review	17	100%	Multiple technologies
Fagerström et al [43], 2017	The role of ICT in nursing practice: an integrative literature review of the Swedish context	Sweden	Integrative literature review	20	100%	Hospital and care institution information systems
Borum [44], 2018	Barriers for hospital-based nurse practitioners utilizing clinical decision support systems: a systematic review	United States	Systematic review	9	78%	Decision support
Koivunen and Saranto [45], 2018	Nursing professionals’ experiences of the facilitators and barriers to the use of telehealth applications: a systematic review of qualitative studies	Finland	Systematic review	25	100%	Telehealth
Ko et al [10], 2018	Nursing home implementation of health information technology: review of the literature finds inadequate investment in preparation, infrastructure, and training	United States	Literature review	46	100%	Hospital and care institution information systems
Li and Cotton [46], 2019	A systematic review of nurses’ perspectives toward the telemedicine intensive care unit: a basis for supporting its future implementation in China?	China	Systematic review	14	94%	Telehealth
Mileski et al [47], 2019	Alarming and/or alerting device effectiveness in reducing falls in long-term care (LTC) facilities? a systematic review	United States	Systematic review	28	78%	Sensor
Mathieson et al [48], 2019	Strategies, facilitators and barriers to implementation of evidence-based practice in community nursing: a systematic mixed-studies review and qualitative synthesis	England	Systematic mixed-studies review	22	100%	Multiple technologies
Surani et al [49], 2019	Role played and strategies employed by managers to support point-of- care nurses’ use and adoption of health information technology: a scoping review	Canada	Scoping review	10	100%	Hospital and care institution information systems
Shiells et al [50], 2019	Electronic patient records as a tool to facilitate care provision in nursing homes: an integrative review	Czech Republic	Integrative review	22	100%	Electronic medical records
Hülsken-Giesler et al [51], 2019	Tracking-systeme bei menschen mit demenz in der stationären langzeitpflege [Tracking systems in people with dementia in long-term care—an integrative review]	Germany	Integrative review	8	100%	Tracking
Konttila et al [52], 2019	Health care professionals’ competence in digitalization: a systematic review	Finland	Systematic review	12	100%	Multiple technologies
Kangasniemi et al [53], 2019	The use of robots and other automated devices in nurses’ work: an integrative review	Finland	Integrative review	25	100%	Robot
Laukka et al [54], 2020	Health care professionals’ experiences of patient-professional communication over patient portals: systematic review of qualitative studies	Finland	Systematic review	13	100%	Assistive device
Matinolli et al [55], 2020	Health and medical device development for fundamental care: scoping review	Finland	Scoping review	19	100%	Multiple technologies
Tolentino and Gephart [56], 2020	State of the science of dimensions of nurses’ user experience when using an electronic health record	United States	Integrative review	26	100%	Electronic medical records
Araujo et al [57], 2020	Clinical decision support systems for pressure ulcer management: systematic review	Portugal	Systematic review	16	94%	Decision support
Arcega et al [58], 2020	The human touch: is modern technology decreasing the value of humanity in patient care?	United States	Literature review	12	86%	Multiple technologies
Brown et al [59], 2020	Issues affecting nurses’ capability to use digital technology at work: an integrative review	Australia	Integrative review	17	100%	Multiple technologies
Joseph et al [60], 2020	The impact of implementing speech recognition technology on the accuracy and efficiency (time to complete) clinical documentation by nurses: a systematic review	Ireland	Systematic review	10	100%	Assistive device
Fraczkowski et al [61], 2020	Nurse workarounds in the electronic health record: an integrative review	United States	Integrative review	33	86%	Electronic medical records
Lewinski et al [9], 2021	Implementing remote triage in large health systems: a qualitative evidence synthesis	United States	Systematic review	32	89%	Decision support
Saab et al [62], 2021	Nurses and nursing students’ attitudes and beliefs regarding the use of technology in patient care: a mixed-method systematic review	Ireland	Mixed-method systematic review	8	78%	Hospital and care institution information systems
Spinewine et al [63], 2021	Interventions to optimize medication use in nursing homes: a narrative review	Belgium	Narrative review	13	100%	Assistive device
Abdellatif et al [64], 2021	Computerized decision support systems for nursing homes: a scoping review	France	Scoping review	24	100%	Decision support
Ferdousi et al [65], 2021	Attitudes of nurses toward clinical information systems: a systematic review and meta-analysis	Iran	Systematic review and meta-analysis	17	100%	Hospital and care institution information systems
Valk-Draad and Bohnet-Joschko [66], 2022	Nursing home-sensitive hospitalizations and the relevance of telemedicine: a scoping review	Germany	Scoping review	16	100%	Telehealth
Setyowati et al [67], 2022	Digital nursing technology to achieve job satisfaction: a systematic review	Indonesia	Systematic review	17	67%	Multiple technologies
Coffetti et al [19], 2022	Individual and team factors influencing the adoption of information and communication technology by nurses: a systematic review	Netherlands	Systematic review	17	100%	Hospital and care institution information systems
Morrison et al [68], 2022	Understanding the use of telehealth in the context of the family nurse partnership and other early years home visiting programmes: a rapid review	England	Rapid review	18	100%	Telehealth
Burgess and Honey [69], 2022	Nurse leaders enabling nurses to adopt digital health: results of an integrative literature review	New Zealand	Integrative literature review	8	100%	Multiple technologies
Bail et al [70], 2022	Using health information technology in residential aged care homes: an integrative review to identify service and quality outcomes	Australia	Integrative review	95	100%	Hospital and care institution information systems
Abdolkhani et al [71], 2022	The impact of digital health transformation driven by COVID-19 on nursing practice: systematic literature review	Australia	Systematic literature review	21	89%	Multiple technologies
Santos et al [72], 2023	Clinical decision support systems for palliative care management: a scoping review	United States	Scoping review	12	100%	Decision support
Huang et al [73], 2023	Intelligent physical robots in health care: systematic literature review	Finland	Systematic literature review	94	100%	Robot
Glanville et al [74], 2023	Handheld computer devices to support clinical decision-making in acute nursing practice: systematic scoping review	Australia	Systematic scoping review	28	100%	Assistive device
O’Connor et al [75], 2023	Artificial intelligence in nursing and midwifery: a systematic review	England	Systematic review	140	78%	Decision support
Wosny et al [76], 2023	Experience of health care professionals using digital tools in the hospital: qualitative systematic review	Switzerland	Systematic review	17	100%	Multiple technologies
Alobayli et al [77], 2023	Electronic health record stress and burnout among clinicians in hospital settings: a systematic review	England	Systematic review	29	89%	Electronic medical records
Ackerhans et al [78], 2024	Exploring the role of professional identity in the implementation of clinical decision support systems—a narrative review	Germany	Narrative review	131	100%	Decision support
Wong et al [79], 2024	Adoption of artificial intelligence–enabled robots in long-term care homes by health care providers: scoping review	Canada	Scoping review	33	86%	Robot
Medina Martin et al [80], 2024	Nurses’ perspectives on ethical aspects of telemedicine. a scoping review	Spain	Scoping review	12	93%	Telehealth
Murali et al [81], 2024	Clinical practice, decision-making, and use of clinical decision support systems in invasive mechanical ventilation: a narrative review	England	Narrative review	29	100%	Decision support
Cachata et al [82], 2024	The integration of information technology in the management and organization of nursing care in a hospital environment: a scoping review	Portugal	Scoping review	9	100%	Hospital and care institution information systems
Chua et al [83], 2024	Facilitators and barriers to implementation of telemedicine in nursing homes: a qualitative systematic review and meta-aggregation	Singapore	Systematic review	81	100%	Telehealth
Galiano et al [84], 2024	Technological innovation for workload allocation in nursing care management: an integrative review	Chile	Integrative review	35	93%	Hospital and care institution information systems
Grechuta et al [85], 2024	Benefits of clinical decision support systems for the management of noncommunicable chronic diseases: targeted literature review	Germany	Literature review	49	100%	Decision support
Shelley et al [86], 2024	Oncology nurses’ experiences of using health information systems in the delivery of cancer care in a range of care settings: a systematic integrative review	Australia	Systematic integrative review	26	94%	Hospital and care institution information systems
Wahyuni et al [87], 2024	Electronic nursing documentation for patient safety, quality of nursing care, and documentation: a systematic review	Saudi Arabia	Systematic review	15	83%	Electronic medical records
Yang et al [88], 2024	Interventions to promote the implementation of pressure injury prevention measures in nursing homes: a scoping review	China	Scoping review	40	100%	Decision support
Zharima et al [89], 2024	What engagement strategies are useful in facilitating the implementation of electronic health records in health care settings? A rapid review of qualitative evidence synthesis using the normalization process theory	South Africa	Rapid review	41	86%	electronic medical records

### Factors Influencing the Implementation and Adoption of DNTs

#### Overview

After analyzing the data, a total of 1031 segments were identified and classified into 52 inductively generated categories (influencing factors) within 6 domains of the NASSS framework.

Of the 1031 segments, the “adopters” domain had the highest number with 432 (41.9%) segments. The “condition” domain primarily identifies factors from the patient’s perspective (Figure 1), but we have made explicit reference to the factors that have an impact on nurses. Similarly, the “wider system” domain does not focus directly on factors from the nurses’ perspective. However, we have decided to include them here for the purposes of completeness. This approach also explains the relatively small number of factors included in these domains compared to the others. There has been no categorization in the seventh domain (“embedding and adaption over time;” Table 2).

It is important to note that the factors described in the included articles were often presented as either facilitators or barriers. For example, in the domain “technology,” the subcategory “functionality” was reported as a barrier in almost all included segments. A lack of user-friendly features or technical issues can hinder the effective implementation of the technology in practice. On the other hand, in the domain “value proposition,” most segments in the “quality of care” subcategory were described as a facilitating factor. However, this does not imply that they are inherently one or the other. In fact, many factors can be both, depending on the context. For example, “functionality” in the domain of “technology” can be seen as a facilitator when it works as intended, but it can become a barrier when it malfunctions. The frequency with which certain factors are mentioned provides insights into which domains and factors are predominantly reported with positive or negative experiences.

**Table 2:** Frequency of factors influencing the implementation and adoption of digital nursing technologies (DNTs).

Domains and Subcategories		Definition	Segments (n=1031^a^), n (%)		Facilitators^b^ (n=569), n (%)		Barriers^b^ (n=462), n (%)	
**Adopters**				432 (41.9)		212 (37.3)		220 (47.6)
	Training	The availability and quality of training programs that support nurses in effectively using DNTs.	58 (13.4)		31 (14.6)		27 (12.3)	
	Workload	The impact of DNTs on workload, including reductions or increases in administrative burden.	54 (12.5)		23 (10.8)		35 (15.9)	
	Technological confidence	The comfort and confidence of nurses in using and troubleshooting DNTs.	42 (9.7)		20 (9.4)		34 (15.4)	
	Efficiency of care	The extent to which DNTs enhance productivity and streamline nursing workflows.	34 (7.9)		19 (9)		23 (10.4)	
	Perceived value	The overall value and benefits that nurses associate with DNTs.	34 (7.9)		25 (11.8)		9 (4.1)	
	Acceptance or positive attitude	The openness of nurses toward DNTs and their willingness to integrate them into care practices.	32 (7.4)		26 (12.3)		8 (3.6)	
	Professional role or identity	The perception of how DNTs influence nursing roles and responsibilities.	32 (7.4)		7 (3.3)		25 (11.4)	
	Autonomy or privacy	The balance between technological support and nurses’ independence in decision-making.	22 (5.1)		7 (3.3)		25 (11.4)	
	Relationship between colleagues	The impact of DNTs on teamwork, collaboration, and interpersonal relationships among staff.	18 (4.2)		19 (9)		3 (1.4)	
	Relationship between nurses and patients	The influence of DNTs on patient-nurse interactions and care quality.	12 (2.8)		13 (6.1)		5 (2.3)	
	New tasks or responsibilities	The introduction of additional responsibilities as a result of DNT implementation.	10 (2.3)		8 (3.8)		4 (1.8)	
	Level of education	The effect of educational background on digital literacy and comfort with DNTs.	10 (2.3)		8 (3.8)		2 (0.9)	
	Level of care experience	The influence of professional experience in the specific care setting on the use of and acceptance toward DNTs.	8 (1.8)		5 (2.4)		5 (2.3)	
	Legal and ethical aspects	The influence of legal regulations and ethical considerations on the adoption of DNTs.	5 (1.2)		—^c^		8 (3.6)	
	Age	The impact of age on the ability and willingness to use DNTs effectively.	3 (0.7)		—		5 (2.3)	
	Gender	The role of gender in influencing perceptions and experiences with DNT adoption.	58 (13.4)		1 (0.5)		2 (0.9)	
**Organization**				213 (20.7)		154 (27.1)		59 (12.8)
	Leadership support	The role of leadership in promoting, implementing, and sustaining the integration of DNTs in nursing practice.	55 (25.8)		49 (31.8)		6 (10.2)	
	Training	The availability and quality of educational programs that prepare nurses to effectively use DNTs.	38 (17.8)		31 (20.1)		7 (11.9)	
	Corporate culture	The values, attitudes, and organizational mindset that influence the acceptance and use of DNTs.	31 (14.6)		23 (14.9)		8 (13.6)	
	Resources	The availability of financial, human, and time-related resources to support the implementation and use of DNTs.	25 (11.7)		7 (4.5)		18 (30.5)	
	Nurses’ participation	The extent to which nurses are involved in decision-making processes regarding the selection, development, and implementation of DNTs.	22 (10.3)		18 (11.7)		4 (6.8)	
	Necessary technical infrastructure	The IT and network infrastructure required for the seamless operation of DNTs, including hardware, software, and support systems.	18 (8.5)		7 (4.5)		11 (18.6)	
	Change-management	The strategies and measures that support the successful introduction and adaptation to DNTs within the organization.	18 (8.5)		15 (9.7)		3 (5.1)	
	Necessary building infrastructure	The physical environment and structural facilities that are required to support the implementation and use of DNTs.	4 (1.9)		3 (1.9)		1 (1.7)	
	Access to technology	The availability and ease of access to DNTs for nurses, ensuring equitable distribution and usability.	2 (0.9)		1 (0.6)		1 (1.7)	
**Technology**				208 (20.2)		75 (13.2)		133 (28.8)
	User experience	The ease of use, intuitiveness, and accessibility of DNTs that influence how effectively nurses can interact with the technology.	63 (30.3)		33 (44)		30 (22.6)	
	Functionality	The ability of DNTs to perform their intended tasks efficiently, without errors or disruptions to workflow.	41 (19.7)		4 (5.3)		37 (27.8)	
	Connectivity and integration	The ability of DNTs to connect with other systems and be seamlessly incorporated into existing workflows.	40 (19.2)		16 (21.3)		24 (18)	
	Reliability and robustness	The dependability of DNTs, including system stability, error resistance, and consistency in functioning.	28 (13.5)		6 (8)		22 (16.5)	
	Health care Provider support	The extent to which health care providers receive assistance in implementing and using DNTs effectively.	14 (6.7)		5 (6.7)		9 (6.8)	
	Participatory design process	The involvement of nurses and other stakeholders in the design and development of DNTs to ensure usability and relevance.	9 (4.3)		6 (8)		3 (2.3)	
	Transparency	The clarity and openness of DNT processes, including decision-making algorithms and data use policies.	7 (3.4)		3 (4)		4 (3)	
	Security and privacy	The protection of patient data and compliance with regulatory standards to ensure confidentiality and trust in DNTs.	6 (2.9)		2 (2.7)		4 (3)	
**Value proposition**				162 (15.7)		125 (22)		37 (8%)
	Quality of care	The extent to which DNTs improve patient safety, reduce errors, and enhance overall care delivery.	44 (27.2)		34 (27.2)		10 (27)	
	Job satisfaction	The influence of DNTs on nurse well-being, job satisfaction, and perceived ease of daily tasks.	31 (19.1)		22 (17.6)		9 (2.7)	
	Efficiency of care	The impact of DNTs on streamlining processes, reducing workload, and optimizing care delivery.	23 (14.2)		21 (16.8)		2 (5.4)	
	Financial return	The economic benefits or costs associated with DNT implementation and long-term sustainability.	10 (6.2)		5 (4)		5 (13.5)	
	Communication	The role of DNTs in enhancing or hindering communication between health care professionals and patients.	10 (6.2)		10 (8)		0 (0)	
	Time	The influence of DNTs on time management, optimizing work processes, and reducing inefficiencies.	8 (4.9)		6 (4.8)		2 (5.4)	
	Evidence	The availability and reliability of research supporting the effectiveness of DNTs in clinical practice.	8 (4.9)		1 (0.8)		7 (18.9)	
	Documentation	The role of DNTs in facilitating accurate, efficient, and accessible documentation of patient care.	8 (4.9)		7 (5.6)		1 (2.7)	
	Knowledge expansion	The potential of DNTs to support continuous learning and professional development for nurses.	7 (4.3)		7 (5.6)		—	
	Accessibility	The ease with which nurses and patients can access and use DNTs, regardless of physical or cognitive limitations.	5 (3.1)		5 (4)		—	
	Autonomy	The extent to which DNTs support or restrict nurses’ decision-making and independence in clinical practice.	4 (2.5)		3 (2.4)		1 (2.7)	
	Coordination	The ability of DNTs to enhance teamwork and communication among health care providers.	4 (2.5)		4 (3.2)		—	
**Condition**				8 (0.8)		—		8 (1.7)
	Diagnosis	The extent to which DNT effectiveness varies depending on patient conditions or diagnoses.	5 (62.5)		—		5 (62.5)	
	Adherence	The willingness and ability of patients to engage with and comply with DNT-supported care protocols.	2 (25)		—		2 (25)	
	Perceived burden for others	The perception that using DNTs places additional strain on family members or caregivers.	1 (12.5)		—		1 (12.5)	
**Wider system**				8 (0.8)		3 (0.5)		5 (1.1)
	Governance involvement	The role of regulatory bodies, policies, and financial models in shaping the adoption and sustainability of DNTs.	5 (62.5)		1 (33.3)		4 (80)	
	Health care costs	The financial implications of DNT implementation, including cost-effectiveness and reimbursement issues.	1 (12.5)		1 (33.3)		—	
	Local context	The influence of regional health care policies, infrastructure, and workforce dynamics on DNT adoption.	1 (12.5)		1 (33.3)		—	
	Environmental setting	The impact of external factors, such as economic conditions, political climate, and technological advancements, on DNT implementation.	1 (12.5)		—		1 (20)	

^a^Absolute and relative values refer to the individual domain in each case.

^b^Specification in the coded segment as a facilitator or a barrier to implementation.

^c^Not applicable.

#### Adopters

This domain focuses on the individuals and groups who will use or be affected by the technology, including their motivation, capacity, and willingness to adopt.

The facilitators and barriers were identified almost equally in this domain. Most factors fell into the categories of “training” (58/432, 13.4%) and “workload” (54/432, 12.5%). Regarding training, nurses’ acceptance and effectiveness in using DNTs were strongly linked to their experience and competence in using these technologies [67]. Adequate training and education were considered essential to improve nurses’ competence and reduce workload [57]. The lack of training and technological knowledge was associated with uncertainty, frustration, and inefficient use of technologies, such as health information systems [70]. The need for specialized training programs tailored to the specific needs of nurses was often highlighted [10]. The “workload” category revealed that while technological innovations can reduce workload by saving time, simplifying tasks, and reducing stress [53], they can also contribute to increased workload by introducing additional technical requirements or redundant documentation tasks, particularly in electronic medical record systems [61]. The “(perceived) value” category (34/432, 7.9%) reflected mostly positive feedback, as nurses reported feeling more confident in managing patient risks and acknowledged that technologies, such as telemedicine [45], improved patient safety and quality of care. However, concerns were raised about the potential for human error and mechanical failure in complex medical devices [33], as well as skepticism about the cost-benefit ratio [31] and the potential impact on quality of care [28].

#### Organization

This domain examines the readiness and capability of the implementing organizations, including infrastructure, leadership, culture, and available resources.

Most segments (154/213, 72.3%) in this domain were described as facilitators. The category “leadership support” (55/213, 25.8%) emphasized the importance of ongoing training and IT support for successful DNT adoption [9]. Leadership engagement and a supportive environment were seen by nurses as crucial for overcoming implementation challenges [48]. Nursing leadership and organizational backing that values nurses’ input help foster a positive adoption culture [28]. Conversely, inadequate IT support and lack of investment in infrastructure and staffing were major barriers [12]. The category “training” (38/213, 17.8%), while also categorized under the “adopters” domain, focuses here on organizational-level structural and resource-related factors. Access to training and continuous support was seen as vital for the successful implementation and use of DNTs. Nurses emphasized the need for dedicated time and sufficient resources for training programs [58]. Effective training strategies, tailored to the diverse needs of staff, including both formal and informal learning opportunities, are crucial for building readiness and capacity among staff [49]. However, barriers such as time constraints, lack of support, and inadequate resources often hinder training efforts, stressing the need for standardized, mandatory training practices [40].

The “resources” category (25/213, 11.7%) included factors such as policy, staffing, and financial constraints. Sufficient physical and technical resources were considered crucial for the sustainable integration of DNTs [49]. The barriers included staff shortages, high costs, time limitations, and the need for dedicated personnel to manage technologies. Moreover, concerns about cost allocation and suboptimal resource distribution were often reported as hindrances to effective implementation [45,63].

#### Technology

This domain involves the features of the technology, including its usability, maturity, complexity, cost, and how it fits into existing workflows.

The assigned segments in this domain were often described as barriers in the included literature. Most (63/208, 30.3%) of the factors fell into the category of “user experience.” While technologies, such as hospital information technology systems [28] and web-based patient-professional communication [54], were positively rated by nurses for their ease of use and intuitive design (functional and technical characteristics of DNTs), facilitating communication and patient care, challenges such as poor usability of clinical decision support systems [35], general technical issues, and time-consuming processes [59] were identified as contributing to nurses’ stress and frustration, ultimately impacting workflow efficiency and quality of patient care, as described, for example, in a review of electronic health records [56]. The category “functionality” (41/208, 19.7%) was predominantly associated with barriers, with only 10% (4/41) of the factors described as facilitators, for example, in the context of clinical information systems [65]. The common challenges included speed, connectivity, and battery life issues associated with technologies such as intelligent physical robots [73] and telehealth solutions [62]. In addition, system malfunctions, software failures, and inefficient documentation processes were frequently reported, particularly in electronic health records [70] and digital nursing information systems [43]. The category “reliability and robustness” (28/208, 13.5%) highlighted the critical importance of reliability in DNT for patient safety. While advances in alarm systems [47] have improved accuracy, challenges such as false alarms, system failures, and unreliable equipment persist, leading to staff fatigue and skepticism about the effectiveness of alarm technologies [36]. Moreover, system downtimes and inaccurate patient data, especially in telehealth applications [67], hindered overall system reliability.

#### Value Proposition

This domain focuses on the perceived benefits of the technology—for developers, health care providers, nurses, and patients.

The assigned segments in this domain were mostly described as facilitators (125/162, 77.2%). The category “quality of care” (44/162, 27.2%) was the most frequently coded factor in this domain. Nurses highlighted the benefits of medication administration technologies [59] in improving patient safety, particularly in reducing treatment errors and adverse events [65]. Several studies indicated that clinical decision support systems [75] and telehealth solutions [46] improved triage efficiency and immediate care delivery, ultimately leading to better patient outcomes [41]. In the “job satisfaction” category (31/162, 19.1%), technology integration was found to contribute to increased job satisfaction, particularly through improved patient monitoring [36] and reduced workload and burden of care, as observed with alarm systems [47]. These benefits allowed nurses to spend more time on direct patient care [50] and enhanced their competency in using DNTs [60]. However, some concerns were raised about the compatibility of certain digital tools with nursing workflows and a perceived lack of understanding of their practical purpose [29]. The “efficiency of care” category (23/162, 14.2%) reflected mixed perceptions. While videoconferencing tools were sometimes perceived to be less effective than face-to-face communication [68], telehealth technologies were widely valued for their potential to enhance care efficiency [83]. In addition, DNTs designed to standardize care processes, such as electronic health records and clinical information systems [35], were seen as beneficial in streamlining care planning, assessments, and shift transitions [50].

#### Condition

This domain refers to the illness or health condition being addressed. It considers its complexity, comorbidities, predictability, and impact on care.

A few factors (8/1031, 0.8%) were assigned to this domain, as many identified themes focused more on organizational or technological issues. Nonetheless, patient-related factors such as adherence—the willingness and ability of patients to consistently engage with digitally supported care—were important considerations. Scheduling consultations posed particular challenges for patients with complex, unstable, or psychosocial conditions. Missed or delayed appointments often reflected deeper difficulties in patient engagement and self-management, which in turn increased the workload for nurses who had to compensate for these gaps [44]. In addition, some nurses expressed concerns about the limitations of web-based consultations for detecting deterioration in patients with complex or high-risk conditions. This raised questions about whether DNTs can reliably support safe care for these vulnerable groups [40].

#### Wider System

This domain considers external influences, such as policies, regulations, standards, and broader sociopolitical and economic factors.

For the same reason as in the “condition” domain, very few factors were assigned in this domain overall (8/1013, 0.8%). Nevertheless, the factors identified are critical to understanding the barriers to successful DNTs implementation and adoption. The category with the most negative factors was “governance involvement” (4/8, 50%), which highlighted several systemic barriers to the integration and widespread adoption of DNTs. A recurring theme was the lack of sufficient funding and unclear reimbursement models, which created uncertainty for health care providers and organizations regarding the financial sustainability of DNT programs [71]. In addition, the lack of regulatory guidelines regarding the integration of DNTs into existing systems and liability for medical errors was another critical barrier. Health care providers expressed concerns about how liability would be handled in the event of errors during virtual care, for example, making them reluctant to fully embrace the technology [71]. Another issue was uncertainty about the return on investment from implementing DNTs, which led to hesitation among stakeholders who were unsure of the long-term benefits. External environmental factors, such as government support and general public opinion about the effectiveness of remote technologies, also influenced the rate of DNTs adoption [34].

## Discussion

### Principal Findings

In this umbrella review, we synthesized evidence from the existing systematic reviews that discuss facilitators and barriers to the implementation and adoption of DNTs. We identified 1031 segments and 52 inductively generated categories within the 6 domains of the NASSS framework, highlighting the key factors influencing implementation. The review shows increasing research interest in DNTs, especially after 2018 (46/65, 71%), with most conducted in Western countries—predominantly the United States (16/65, 25%). The research focused on electronic health records, clinical decision support systems, and telehealth, while robotics and automation received less attention.

Nurses represent nearly 45% of the global health workforce and frequently act as the link between patients, technologies, and organizational structures. Their acceptance and engagement are therefore critical to successful implementation and adoption. While prior reviews take a general or physician-centered approach [91,92], our nursing-specific focus uncovers unique facilitators and barriers—such as professional identity, autonomy, and relational care—that are commonly overlooked. We believe these insights are essential not only for enhancing DNT implementation in nursing practice but also for providing valuable guidance for developers, policy makers, and health care teams working toward sustainable adoption.

We categorized the identified factors into overarching themes instead of focusing on specific technologies or settings, enabling synthesis across contexts while preserving nuance. However, certain technologies (eg, direct patient interaction vs infrastructure tools) pose unique challenges. Nurses’ needs vary by care setting, influencing how often facilitators and barriers appear across NASSS domains. Some factors even act as both facilitators and barriers. This uneven distribution suggests some domains may be more central to implementation than others.

To increase the likelihood of sustainable implementation and adoption of DNTs, it is recommended that the specific needs of the technology and the setting be considered [17-20], as highlighted in the “technology” and “organization” domains, where usability, infrastructure, and contextual factors varied significantly. Abell et al [93], in a recent scoping review, explored the barriers and facilitators to implementing computerized clinical decision support systems in hospitals. Their review included a broad range of health care professionals, such as physicians, physician assistants, nurses, and pharmacists. Although their focus was limited to one type of technology, their findings—structured using the NASSS framework—align with ours. This supports both the applicability of the NASSS framework in analyzing implementation challenges and the presence of generic influencing factors relevant across different DNTs. Tan et al [94], in another scoping review, examined the factors influencing the implementation and adoption of telehealth in nursing home settings. They identified critical factors, such as availability of technical infrastructure, staff training and acceptance, financial considerations, and leadership support. The authors highlight the complexity of implementing telehealth in care settings and the need to address these determinants to ensure successful implementation. In addition, in the context of informal dementia care, the results of a comprehensive review by Bastoni et al [95] on the factors influencing the implementation of eHealth technologies show similar findings to our work. The results of these studies [93-95] show that the factors influencing the implementation and adoption of DNTs found in our study can be applied (to a limited extent) to other health care professions and settings. This perspective is supported by several studies in the literature. For instance, Borges do Nascimento et al [92] identified key barriers, such as inadequate infrastructure, increased workload, insufficient training, and legal-ethical concerns. Conversely, they highlighted robust institutional training, governmental support, and involving health care professionals in technology development as crucial facilitators—especially when technologies are intuitively designed and their benefits are evident.

Most factors (432/1031, 41.9%) identified in the included studies relate to the “adopters” domain, underscoring the importance of addressing these barriers and facilitators. Adequate training and education on DNT use are crucial for the successful implementation. Similar conclusions have been drawn in studies on digital transformation in nursing education [11]. Current research highlights several issues: a lack of objective tools to assess digital competencies [96,97], insufficient training that addresses varying competencies [98,99], and limited implementation knowledge [6,99]. The NASSS framework helped structure these findings across individual, organizational, and systemic levels, enabling a holistic view of training-related challenges. Well-trained nurses can help address these issues—an approach supported by recent reviews as a key to overcoming barriers and fostering DNT adoption [100].

Our review highlighted the importance of structural and resource-related factors within health care organizations in shaping nurses’ experiences with technology implementation. Leadership support was consistently identified as a key facilitator. Nurses emphasized the role of organizational leaders who ensured ongoing education, IT support, and a supportive work environment in fostering a positive culture for adoption. Nursing leadership, in particular, was seen as crucial in addressing challenges, advocating for staff needs, and enabling integration into clinical routines. In contrast, lack of support, poor IT infrastructure, and staffing shortages were frequently cited as major barriers. These findings align with the previous research emphasizing the necessity of organizational commitment for the sustained adoption of DNTs [101].

The value of DNTs largely depends on their impact on the quality of care [59]. Manufacturer promises often do not reflect nurses’ perceptions. Acceptance increases when nurses are involved in the design and implementation. For instance, bed-exit systems aim to prevent falls but may cause alarm fatigue [13], illustrating the need for usability that fits nursing workflows. Misalignment often stems from development driven by biomedical and economic perspectives, overlooking nursing priorities [14]. Nurses appear to prioritize tools and systems that improve patient outcomes, streamline processes, and ultimately contribute to better patient care. Conversely, when technology complicates workflow or detracts from direct patient interaction, its perceived value diminishes, regardless of the other benefits it may provide (eg, improved efficiency). Therefore, the successful implementation and adoption of technology in the care setting depends on its ability to positively impact the quality of care provided [14]. Integrating participatory design approaches (co-design) in the development phase could help ensure that new digital tools meet the practical needs of nurses and enhance their usability [102].

In our study, the “condition” domain yielded little information, which aligns with our nursing-specific focus—most relevant factors were classified under the “adopters” domain. Similarly, few aspects were related to the “wider system” domain, and none were associated with the “embedding over time” domain, consistent with the findings of Bastoni et al [95]. This gap reflects the dominance of short-term, small-scale studies that often lack structured implementation frameworks. However, understanding how DNTs are sustained, adapted, or discontinued over time is essential for assessing their long-term impact. Without longitudinal perspectives, true integration of DNTs into nursing practice remains unclear. This requires targeted funding, stronger academic-practice partnerships, and wider use of frameworks that address sustainability explicitly.

Political and regulatory factors play an important role in shaping the digital transformation of health care. The lack of clear regulatory guidelines, reimbursement policies, and national funding programs can hinder the widespread adoption of DNTs. Policy makers should focus on creating standardized frameworks that facilitate implementation while ensuring data security, liability clarity, and financial incentives for health care institutions [103].

### Implications for Practice and Research

This review highlights several critical factors influencing the successful implementation and adoption of DNTs in health care settings, offering clear directions for both practice and research (Textbox 2).

**Implications for practice and research.**

**Implications for practice**

- Ensure strong leadership support to foster a positive organizational culture for digital nursing technology (DNT) implementation and adoption.
- Provide tailored training programs that meet the specific digital competency needs of nurses.
- Guarantee adequate IT infrastructure, staffing, and ongoing technical support.
- Promote collaboration among key stakeholders (eg, nurses, nursing leadership, IT specialists, and administrators) during all phases of implementation.
- Integrate DNTs in a way that aligns with nursing workflows and supports professional autonomy and relational care.

**Implications for research**

- Apply structured implementation frameworks (eg, NASSS [nonadoption, abandonment, scale-up, spread, and sustainability]) in future studies to better understand how various factors influence DNT adoption across technologies, settings, and user groups.
- Conduct longitudinal research to explore how DNTs are sustained, adapted, or discontinued over time and across different institutional contexts.
- Develop practical, user-friendly tools to support researchers and practitioners during the implementation process, as current instruments (eg, NASSS Complexity Assessment Tool [104]) often lack ease of use.

### Strengths and Limitations

Using a sensitive search strategy, we synthesized a substantial knowledge base through a comprehensive examination of reviews. Furthermore, the strength of our review lies in the use of the NASSS framework as a deductive categorization system [14]. However, it is imperative to acknowledge specific limitations. Our findings are context-specific to formal care, and their applicability to other implementation contexts may vary. While there is overlap with other professions and settings, it is important to note that different technologies present their own unique challenges that may not have been fully captured in this broader analysis. Although common themes were identified across technologies, each technology may present its own set of barriers and facilitators that were not always addressed in this review, which may affect the applicability of the findings to specific technology contexts.

Another limitation is that although we searched 4 highly relevant databases (PubMed, CINAHL, Cochrane Library, and Business Source Premier), we cannot ensure that we included all existing reviews in our study. The selection of these databases was based on their relevance to the research topic and their coverage of health sciences, systematic reviews, and business literature. However, we acknowledge that the exclusion of broader databases, such as Scopus or Google Scholar, and other discipline-specific databases, such as PsycINFO or Embase, may have limited the scope of our search and resulted in the omission of relevant studies. In addition, we included only German and English manuscripts, which may introduce a potential regional bias by excluding studies published in other languages, such as French, Chinese, or Spanish. Although this decision was based on the linguistic expertise of the research team, we acknowledge that a fully comprehensive picture of the global landscape of DNT adoption and implementation could not be achieved, and it may limit the transferability of our findings to other cultural and linguistic contexts.

The quality of the included studies was assessed using the Joanna Briggs Institute Critical Appraisal Checklist for Systematic Reviews and Research Syntheses [27]. As most studies were narrative reviews with a qualitative focus, purely quantitative quality metrics were less applicable. Instead, we prioritized methodological transparency, including clear research questions, a rigorous study selection process, and transparent analysis. Given the qualitative nature of these reviews, we focused on coherence, relevance, and clarity in the synthesis of findings rather than statistical measures. After careful consideration, we included all reviews in the synthesis while ensuring transparency in the quality assessment. However, the varying quality of the included reviews may have influenced the strength and reliability of the synthesized findings.

Finally, it is important to acknowledge the potential publication bias, as only published reviews were included in our synthesis. Although we made a high effort (including a search update) to ensure a comprehensive search, the exclusion of unpublished or gray literature may have led to an overrepresentation of certain outcomes and potentially biased the results.

### Conclusions

This umbrella review provides a comprehensive synthesis of the facilitators and barriers to the implementation and adoption of DNTs. It highlights the complex interplay of organizational, individual, and systemic factors that influence the successful integration of new technologies into care processes. Leadership support, adequate training, and alignment with care needs emerge as key enablers, while insufficient resources, infrastructure limitations, and workflow incompatibilities remain substantial barriers. The findings underscore the importance of addressing both the specific technology requirements and the unique needs of the health care environment to ensure the successful implementation of DNTs. Future research should focus on long-term studies and the development of practical tools to support hospital stakeholders—such as nurses, management, and IT departments—in effectively adopting DNTs. This will help to sustainably embed DNTs in nursing practice and increase their long-term impact.

## Appendix

Multimedia Appendix 1 Digital nursing technology categories and definitions.

Multimedia Appendix 2 Search string.

Multimedia Appendix 3 Quality of the included studies.

Multimedia Appendix 4 PRISMA checklist.

## Abbreviations

DNT: digital nursing technology
MeSH: Medical Subject Headings
NASSS: nonadoption, abandonment, scale-up, spread, and sustainability
PICO: population, intervention, comparison, outcome
PRISMA: Preferred Reporting Items for Systematic Reviews and Meta-Analyses

## Data Availability

The datasets generated and analyzed during this study are available from the corresponding author upon reasonable request.
