# Supplementary material for: Factors Influencing the Implementation and Adoption of Digital Nursing Technologies: Systematic Umbrella Review
Source: J Med Internet Res. 2025 Jul 31;27:e64616. doi: 10.2196/64616 (PMC12355146; doi:10.2196/64616)
Supplement: Multimedia Appendix 1 [file jmir_v27i1e64616_app1.docx]

Factors Influencing Implementation and Adoption of Digital Nursing Technologies – Systematic Umbrella Review

Multimedia Appendix 1: Digital Nursing Technology Categories and Definitions

Digital nursing technologies (DNTs) are tools designed to help nurses provide efficient, high-quality care by facilitating rapid decision-making (e.g., decision support systems for adherence to clinical guidelines) or complementing nursing tasks with technological solutions (e.g., robots that assess vital signs before consultations or sensors that detect bed-exit events to prevent falls). These technologies serve multiple purposes, including enhancing patient safety, improving workflow efficiency, and reducing the physical and cognitive burden on nurses. DNTs can function as stand-alone tools for individual nurses or be embedded in broader organizational care processes, such as integrated electronic health record systems or automated medication dispensing systems. By addressing specific clinical or operational needs, DNTs aim to improve care delivery, optimize resource use and support nurses in managing complex care environments.

Based on Krick et al [1], the following categorization of technologies is used:

Table 1. Digital nursing technology categories and definitions.

| Category | Definitions |
| --- | --- |
| Assistive Device | Assistive Devices assist or support a nurse in performing a particular task and are enhanced with digital technology, i.e., are digitally connected or equipped with sensor technology. |
| Decision Support | Decision support systems are software solutions that link individual patient data (input) with treatment guidelines and a recommendation (output) to be delivered to a person in charge of care. |
| EHR/EMR | Electronic health records (EHR) and electronic medical records (EMR) are digital records of patient related health information. EMR refers to patient data that is stored and exchanged inside an institution, mostly a hospital. The main focus of the EHR is the capability to exchange information between two systems. |
| HIS | Hospital/Care Institution Information Systems (HIS) collect, store, manage and transmit data in hospitals or other care institutions. They can comprise operational management systems, EMR and/or other organizational systems. |
| Monitoring | Monitoring technologies are complex and analytical technologies to monitor patient, caregiver or organizational relevant data over a period. They often integrate sensors but are more complex than single sensor solutions. |
| Multiple Technologies | Interventions/studies that include technologies from different technology categories. |
| Robot | Robots are machines that interact with their physical environment by sensors, actuators and information technology. This includes social assistive robots, physical assistive robots and complex robotic systems. |
| Sensor | Sensors measure physical or chemical properties and are used to assess, e.g., behavior, movements or odors. They are often used to control/trigger other devices like pumps or alarm systems. |
| Telehealth | Telehealth refers to the use of digital technologies to provide remote healthcare services, including consultations, monitoring, and the exchange of medical information. |
| Tracking | Tracking technologies locate people or objects. |

References

1. Krick T, Huter K, Domhoff D, Schmidt A, Rothgang H, Wolf-Ostermann K. Digital technology and nursing care: a scoping review on acceptance, effectiveness and efficiency studies of informal and formal care technologies. BMC Health Serv Res 2019;19(1):400. PMID:31221133
