# Supplementary material for: Factors Influencing the Implementation and Adoption of Digital Nursing Technologies: Systematic Umbrella Review
Source: J Med Internet Res. 2025 Jul 31;27:e64616. doi: 10.2196/64616 (PMC12355146; doi:10.2196/64616)
Supplement: Multimedia Appendix 2 [file jmir_v27i1e64616_app2.docx]

Factors Influencing Implementation and Adoption of Digital Nursing Technologies – Systematic Umbrella Review

Multimedia Appendix 2: Search Strategy

[The updated research was carried out using the same search strings and included publications up to January 2025.]

Table 1: Preliminary search on MEDLINE via PubMed (21.04.2023).

| PIO | # | Search string | Results |
| --- | --- | --- | --- |
| Population | | | |
|  | 1 | nurs*[Title/Abstract] | 526,190 |
|  | 2 | caregiv*[Title/Abstract] | 95.286 |
|  | 3 | nursing staff [Title/Abstract] | 14.485 |
|  |  |  |  |
|  | 4 | OR/#1-#3 | **608,496** |
| Intervention | | | |
|  | 5 | digital technology [Title/Abstract] | 2,768 |
|  | 6 | technol*[Title/Abstract] | 646,594 |
|  | 8 | digital*[Title/Abstract] | 195,513 |
|  | 9 | Telemedicine [MeSH Terms] | 43,735 |
|  | 10 | Diffusion of Innovation [MeSH Terms] | 21,448 |
|  | 11 | Change Management [Title/Abstract] | 1,956 |
|  | 12 | Nursing Informatics [MeSH Terms] | 1,630 |
|  | 13 | Electronic Health Records [MeSH Terms] | 27,254 |
|  | 14 | Medical Order Entry Systems [MeSH Terms] | 2,449 |
|  | 15 | innovat*[Title/Abstract] | 188,178 |
|  | 16 | ehealth[Title/Abstract] | 9,325 |
|  | 17 | e-health [Title/Abstract] | 4,342 |
|  | 18 | electronic health [Title/Abstract] | 29,801 |
|  | 19 | telehealth [Title/Abstract] | 13,076 |
|  | 20 | tele-health [Title/Abstract] | 282 |
|  | 21 | telemonitor [Title/Abstract] | 36 |
|  | 22 | medical information [Title/Abstract] | 10,657 |
|  | 23 | medical order [Title/Abstract] | 232 |
|  | 24 | ICT [Title/Abstract] | 7,648u |
|  | 25 | robot* [Title/Abstract] | 67,890 |
|  | 26 | informatics [Title/Abstract] | 19,140 |
|  | 27 | sensor [Title/Abstract] | 139,067 |
|  | 28 | monitor* [Title/Abstract] | 975,762 |
|  | 29 | assistive device [Title/Abstract] | 1,158 |
|  | 30 | decision support [Title/Abstract] | 21,792 |
|  |  |  |  |
|  | 31 | OR#5-#30 | **3,309,044** |
| Outcome | | | |
|  | 32 | introduc*[Title/Abstract] | 1,179,678 |
|  | 33 | establish*[Title/Abstract] | 1,442,448 |
|  | 34 | process*[Title/Abstract] | 2,718,554 |
|  | 35 | optimi*[Title/Abstract] | 568,992 |
|  | 36 | facilitat*[Title/Abstract] | 674,152 |
|  | 37 | benefi*[Title/Abstract] | 1,181,518 |
|  | 38 | success [Title/Abstract] | 340,418 |
|  | 39 | failure [Title/Abstract] | 812,074 |
|  | 40 | barrier*[Title/Abstract] | 392,751 |
|  | 41 | Attitude [MeSH Term] | 633,035 |
|  | 42 | Attitude to computers [MeSH Terms] | 4,889 |
|  | 43 | Motivation [MeSH Term] | 192,117 |
|  | 44 | user*[Title/Abstract] | 253,684 |
|  | 45 | inpatient [MeSH Term] | 160,664 |
|  | 46 | Implementation Science [Title/Abstract] | 5,045 |
|  | 47 | adopt*[Title/Abstract] | 330,213 |
|  | 48 | accept*[Title/Abstract] | 548,309 |
|  | 49 | belie*[Title/Abstract] | 347,799 |
|  | 50 | motivat*[Title/Abstract] | 172,228 |
|  | 51 | assimil*[Title/Abstract] | 32,823 |
|  | 52 | percept*[Title/Abstract] | 380,832 |
|  | 53 | attitude*[Title/Abstract] | 3,186 |
|  | 54 | readiness [Title/Abstract] | 22,071 |
|  |  |  |  |
|  | 55 | OR/#5-#28 | **15,958,588** |
|  |  |  |  |
|  | 32 | #4 AND #31 AND #55 + Filters: Review, Systematic Review, English, German, from 2006 - 2023 | **3,145** |

Table 2: Preliminary search on CINAHL, Business Source Premier via EBSCO (23.04.2023)

| PIO | # | Search string | Results |
| --- | --- | --- | --- |
| Population | | | |
|  | 1 | TI (nurs*) OR AB (nurs*) |  |
|  | 2 | TI (caregiver) OR AB (caregiver) |  |
|  | 3 | TI (nursing staff) OR AB (nursing staff) |  |
|  |  |  |  |
|  | 4 | OR/#1-#3 | **1,159,447** |
| Intervention | | | |
|  | 5 | TI (digital technology) OR AB (digital technology) |  |
|  | 6 | TI (technol*) OR AB (technol*) |  |
|  | 7 | TI (digital*) OR AB (digital) |  |
|  | 8 | MH (Telemedicine) OR AB (Telemedicine) |  |
|  | 9 | MH (Diffusion of Innovation) OR AB (Diffusion of Innovation) |  |
|  | 11 | MH (Change Management) OR AB (Change Management) |  |
|  | 12 | MH (Nursing Informatics) OR AB (Nursing Informatics) |  |
|  | 13 | MH (Electronic Health Records) OR AB (Electronic Health Records) |  |
|  | 14 | MH (Medical Order Entry Systems) OR AB (Medical Ordner Entry Systems) |  |
|  | 15 | TI (innovat*) OR AB (innovat*) |  |
|  | 16 | TI (ehealth) OR AB (ehealth) |  |
|  | 17 | TI (e-health) OR AB (e-health) |  |
|  | 18 | TI (electronic health) OR AB (electronic health) |  |
|  | 19 | TI (telehealth) OR AB (telehealth) |  |
|  | 20 | TI (tele-health) OR AB (tele-health) |  |
|  | 21 | TI (telemonitor) OR AB (telemonitor) |  |
|  | 22 | TI (medical information) OR AB (medical information) |  |
|  | 23 | TI (medical order) OR AB (medical order) |  |
|  | 24 | TI (ICT) OR AB (ICT) |  |
|  | 25 | TI (robot*) OR AB (robot*) |  |
|  | 26 | TI (informatics) OR AB (informatics) |  |
|  | 27 | TI (sensor) OR AB (sensor) |  |
|  | 28 | TI (monitor*) OR AB (monitor*) |  |
|  | 29 | TI (assistive device) OR AB (assistive device) |  |
|  | 30 | TI (decision support) OR AB (decision support) |  |
|  |  |  |  |
|  | 31 | OR/#5-#30 | **4,216,993** |
|  |  |  |  |
| Outcome | | | |
|  | 32 | TI (introduc*) OR AB (introduc*) |  |
|  | 33 | TI (establish*) OR AB (establish*) |  |
|  | 34 | TI (evalu*) OR AB (evalu*) |  |
|  | 35 | TI (process*) OR AB (process*) |  |
|  | 36 | TI (optimi*) OR AB (optimi*) |  |
|  | 37 | TI (facilitat*) OR AB (facilitate*) |  |
|  | 38 | TI (benefi*) OR AB (benefi*) |  |
|  | 39 | TI (develop*) OR AB (develop*) |  |
|  | 40 | TI (success) OR AB (success) |  |
|  | 41 | TI (failure) OR AB (failure) |  |
|  | 42 | TI (barrier*) OR AB (barrier*) |  |
|  | 43 | TI (attitude) OR AB (attitude) |  |
|  | 44 | TI (attitude to computers) OR AB (attitude to computers) |  |
|  | 45 | TI (challeng*) OR AB (challeg*) |  |
|  | 46 | TI (motivation) OR AB (motivation |  |
|  | 47 | TI (user*) OR AB (user*) |  |
|  | 48 | TI (inpatient) OR AB (inpatient) |  |
|  | 49 | MH (Implementation Science) OR AB (Implementation Science) |  |
|  | 50 | TI (adopt*) OR AB (adopt*) |  |
|  | 51 | TI (accept*) OR AB (accept*) |  |
|  | 52 | TI (belie*) OR AB (belie*) |  |
|  | 53 | TI (motivat*) OR AB (motivat*) |  |
|  | 54 | TI (assimil*) OR AB (assimil*) |  |
|  | 55 | TI (percept*) OR AB (percept*) |  |
|  | 56 | TI (attitude*) OR AB (attitude*) |  |
|  | 57 | TI (readiness) OR AB (readiness) |  |
|  |  |  |  |
|  | 58 | OR/#32-#57 | **1,996,996** |
|  |  |  |  |
|  | 59 | #4 AND #31 AND #58 + Filters: Review, Systematic Review, English, German, from 2006 - 2023 | **1,299** CINAHL &  **38** Business Source Premier |

Table 3: Preliminary search on Cochrane Library (23.04.2023)

| PIO | # | Search string | Results |
| --- | --- | --- | --- |
| Population | | | |
|  | 1 | nurs* [ti,ab,kw] |  |
|  | 2 | caregiver [ti,ab,kw] |  |
|  | 3 | nursing staff [ti,ab,kw] |  |
|  |  |  |  |
|  | 4 | OR/#1-#3 | **851** |
| Intervention | | | |
|  | 5 | digital technology [ti,ab,kw] |  |
|  | 6 | technol* [ti,ab,kw] |  |
|  | 7 | digital* [ti,ab,kw] |  |
|  | 8 | Telemedicine [ti,ab,kw] |  |
|  | 9 | Diffusion of Innovation [ti,ab,kw] |  |
|  | 11 | Change Management [ti,ab,kw] |  |
|  | 12 | Nursing Informatics [ti,ab,kw] |  |
|  | 13 | Electronic Health Records [ti,ab,kw] |  |
|  | 14 | Medical Order Entry Systems [ti,ab,kw] |  |
|  | 15 | innovat* [ti,ab,kw] |  |
|  | 16 | ehealth [ti,ab,kw] |  |
|  | 17 | e-health [ti,ab,kw] |  |
|  | 18 | electronic health [ti,ab,kw] |  |
|  | 19 | telehealth [ti,ab,kw] |  |
|  | 20 | tele-health [ti,ab,kw] |  |
|  | 21 | telemonitor [ti,ab,kw] |  |
|  | 22 | medical information [ti,ab,kw] |  |
|  | 23 | medical order [ti,ab,kw] |  |
|  | 24 | ICT [ti,ab,kw] |  |
|  | 25 | robot* [ti,ab,kw] |  |
|  | 26 | informatics [ti,ab,kw] |  |
|  | 27 | sensor [ti,ab,kw] |  |
|  | 28 | monitor* [ti,ab,kw] |  |
|  | 29 | assistive device [ti,ab,kw] |  |
|  | 30 | decision support [ti,ab,kw] |  |
|  |  |  |  |
|  | 31 | Or/#5-#30 | **5665** |
|  |  |  |  |
| Outcome | | | |
|  | 32 | introduc* [ti,ab,kw] |  |
|  | 33 | establish* [ti,ab,kw] |  |
|  | 34 | evalu* [ti,ab,kw] |  |
|  | 35 | process* [ti,ab,kw] |  |
|  | 36 | optimi* [ti,ab,kw] |  |
|  | 37 | facilitat* [ti,ab,kw] |  |
|  | 38 | benefi* [ti,ab,kw] |  |
|  | 39 | develop* [ti,ab,kw] |  |
|  | 40 | success [ti,ab,kw] |  |
|  | 41 | failure [ti,ab,kw] |  |
|  | 42 | barrier* [ti,ab,kw] |  |
|  | 43 | attitude [ti,ab,kw] |  |
|  | 44 | attitude to computers [ti,ab,kw] |  |
|  | 45 | challeng* [ti,ab,kw] |  |
|  | 46 | motivation [ti,ab,kw] |  |
|  | 47 | user* [ti,ab,kw] |  |
|  | 48 | inpatient [ti,ab,kw] |  |
|  | 49 | Implementation Science [ti,ab,kw] |  |
|  | 50 | adopt* [ti,ab,kw] |  |
|  | 51 | accept* [ti,ab,kw] |  |
|  | 52 | belie* [ti,ab,kw] |  |
|  | 53 | motivat* [ti,ab,kw] |  |
|  | 54 | assimil* [ti,ab,kw] |  |
|  | 55 | percept* [ti,ab,kw] |  |
|  | 56 | attitude* [ti,ab,kw] |  |
|  | 57 | readiness [ti,ab,kw] |  |
|  |  |  |  |
|  | 58 | OR/#5-#31 | **3190** |
|  |  |  |  |
|  | 59 | #4 AND #31 AND #58 + Filters: From 2006 - 2023 | **258** |
