# Supplementary material for: Factors Influencing the Implementation and Adoption of Digital Nursing Technologies: Systematic Umbrella Review
Source: J Med Internet Res. 2025 Jul 31;27:e64616. doi: 10.2196/64616 (PMC12355146; doi:10.2196/64616)
Supplement: Multimedia Appendix 3 [file jmir_v27i1e64616_app3.docx]

Factors Influencing Implementation and Adoption of Digital Nursing Technologies – Systematic Umbrella Review

Multimedia Appendix 3: Quality of the Included Studies [1]

| Quality criteria  Author  (Year) | 1.  Is the review question clearly and explicitly stated? | 2.  Were the inclusion criteria appropriate for the review question? | 3.  Was the search strategy appropriate? | 4.  Were the sources and resources used to search for studies adequate? | 5.  Were the criteria for appraising studies appropriate?* | 6.  Was critical appraisal conducted by two or more reviewers independently?* | 7.  Were the methods used to combine studies appropriate? | 8. Was the likelihood of publication bias assessed?* | 9.  Were recommendations for policy and/or practice supported by the reported data? | 10.  Were the specific directives for new research appropriate? | Result |
| --- | --- | --- | --- | --- | --- | --- | --- | --- | --- | --- | --- |
| Abdellatif et al [2], 2021 | Met | Met | Met | Met | NA | NA | Met | NA | Met | Met | 7/7  (100%) |
| Abdolkhani et al [3], 2022 | Met | Met | Met | Met | Unclear | Unclear | Met | Not met | Met | Met | 8/9  (89%) |
| Ackerhans et al [4], 2024 | Met | Met | Met | Met | NA | NA | Met | NA | Met | Met | 7/7  (100%) |
| Alobayli et al [5], 2023 | Met | Met | Met | Met | Met | Not met | Met | Not met | Met | Met | 8/9  (89%) |
| Araujo et al [6], 2020 | Met | Met | Met | Met | Met | Unclear | Met | Not met | Met | Met | 8.5/9  (94%) |
| Arcega et al [7], 2020 | Met | Met | Met | Met | NA | NA | Met | NA | Met | Not met | 6/7  (86%) |
| Bail et al [8], 2022 | Met | Met | Met | Met | NA | NA | Met | NA | Met | Met | 7/7  (100%) |
| Borum et al [9], 2018 | Met | Met | Met | Met | Not met | Not met | Met | Not met | Met | Met | 7/9  (78%) |
| Brown et al [10], 2020 | Met | Met | Met | Met | NA | NA | Met | NA | Met | Met | 7/7  (100%) |
| Burgess and Honey [11], 2022 | Met | Met | Met | Met | NA | NA | Met | NA | Met | Met | 7/7  (100%) |
| Cachata et al [12], 2024 | Met | Met | Met | Met | NA | NA | Met | NA | Met | Met | 7/7  (100%) |
| Chua et al [13], 2024 | Met | Met | Met | Met | Met | Met | Met | Not met | Met | Met | 9/9  (100%) |
| Coffetti et al [14], 2022 | Met | Met | Met | Met | Met | Met | Met | Not met | Met | Met | 9/9  (100%) |
| Dos Santos et al [15], 2023 | Met | Met | Met | Met | NA | NA | Met | NA | Met | Met | 7/7  (100%) |
| Fagerström et al [16], 2017 | Met | Met | Met | Met | NA | NA | Met | NA | Met | Met | 7/7  (100%) |
| Ferdousi et al [17], 2021 | Met | Met | Met | Met | Met | Met | Met | Not met | Met | Met | 9/9  (100%) |
| Fraczkowski et al [18], 2020 | Met | Unclear | Unclear | Met | NA | NA | Met | NA | Met | Met | 6/7  (86%) |
| Galiano et al [19], 2024 | Met | Met | Met | Met | NA | NA | Met | NA | Met | Unclear | 6.5/7  (93%) |
| Glanville et al [20], 2023 | Met | Met | Met | Met | NA | NA | Met | NA | Met | Met | 7/7  (100%) |
| Grechuta et al [21], 2024 | Met | Met | Met | Met | NA | NA | Met | NA | Met | Met | 7/7  (100%) |
| Huang et al [22], 2023 | Met | Met | Met | Met | Met | Met | Met | Not met | Met | Met | 9/9  (100%) |
| Hülsken-Gießler et al [23], 2019 | Met | Met | Met | Met | NA | NA | Met | NA | Met | Met | (7/7)  100% |
| Joseph et al [24], 2020 | Met | Met | Met | Met | Met | Met | Met | Not met | Met | Met | 9/9  (100%) |
| Kangasniemi et al [25], 2019 | Met | Met | Met | Met | NA | NA | Met | NA | Met | Met | 7/7  (100%) |
| Kaye [26], 2017 | Met | Met | Met | Met | NA | NA | Met | NA | Met | Met | 7/7  (100%) |
| Ko et al [27], 2018 | Met | Met | Met | Met | NA | NA | Met | NA | Met | Met | 7/7  (100%) |
| Koivunen and Saranto [28], 2017 | Met | Met | Met | Met | Met | Met | Met | Not met | Met | Met | 9/9  (100%) |
| Kontilla et al [29], 2019 | Met | Met | Met | Met | Met | Met | Met | Not met | Met | Met | 9/9  (100%) |
| Kosse et al [30], 2013 | Met | Met | Met | Met | Met | Met | Met | Not met | Met | Met | 9/9  (100%) |
| Kumar et al [31], 2013 | Met | Met | Met | Met | NA | NA | Met | NA | Unclear | Unclear | 6/7  (86%) |
| Laukka et al [32], 2020 | Met | Met | Met | Met | Met | Met | Met | Not met | Met | Met | 9/9  (100%) |
| Lewinski et al [33], 2021 | Met | Met | Unclear | Met | Met | Unclear | Met | Not met | Met | Met | 8/9  (89%) |
| Li and Cotton [34], 2019 | Met | Met | Met | Met | Met | Unclear | Met | Not met | Met | Met | 8.5/9  (94%) |
| Medina Martin et al [35], 2024 | Met | Met | Met | Met | NA | NA | Met | NA | Met | Unclear | 6.5/7  (93%) |
| Matinolli et al [36], 2020 | Met | Met | Met | Met | NA | NA | Met | NA | Met | Met | 7/7  (100%) |
| Mathieson et al [37], 2019 | Met | Met | Met | Met | Met | Met | Met | Not met | Met | Met | 9/9  (100%) |
| Mileski et al [38], 2017 | Unclear | Unclear | Met | Met | Not met | Not met | Met | Not met | Met | Not met | 5/9  (56%) |
| Mileski et al [39], 2019 | Met | Met | Met | Met | Not met | Not met | Met | Not met | Met | Met | 7/9  (78%) |
| Morrison et al [40], 2022 | Met | Met | Met | Met | NA | NA | Met | NA | Met | Met | 7/7  (100%) |
| Murali et al [41], 2024 | Met | Met | Met | Met | NA | NA | Met | NA | Met | Met | 7/7  (100%) |
| O’Connor et al [42], 2022 | Met | Met | Met | Met | Not met | Not met | Met | Not met | Met | Met | 7/9  (78%) |
| Penny et al [43], 2017 | Met | Met | Met | Met | Met | Met | Met | Not met | Met | Met | 9/9  (100%) |
| Piscotty and Kalisch [44], 2014 | Met | Met | Met | Met | NA | NA | Met | NA | Not met | Met | 6/7  (86%) |
| Radhakrishnan et al [45], 2016 | Met | Met | Met | Met | Met | Not met | Met | Not met | Met | Met | 8/9  (89%) |
| Saab et al [46], 2021 | Met | Met | Met | Met | Met | Not met | Met | Not met | Met | Not met | 7/9  (78%) |
| San et al [47], 2012 | Met | Met | Met | Met | Met | Met | Met | Not met | Met | Met | 9/9  (100%) |
| Setyowati et al [48], 2022 | Met | Met | Met | Met | Not met | Not met | Met | Not met | Met | Not met | 6/9  (67%) |
| Shelley et al [49], 2024 | Met | Met | Met | Met | Met | Met | Met | Not met | Met | Unclear | 8.5/9  (94%) |
| Shiells et al [50], 2019 | Met | Met | Met | Met | NA | NA | Met | NA | Met | Met | 7/7  (100%) |
| Spinewine et al [51], 2021 | Met | Met | Met | Met | NA | NA | Met | NA | Met | Met | 7/7  (100%) |
| Stevenson et al [52], 2010 | Met | Unclear | Met | Met | NA | NA | Met | NA | Met | Met | 6,5/7  (93%) |
| Strudwick [53], 2015 | Met | Met | Met | Met | NA | NA | Met | NA | Met | Met | 7/7  (100%) |
| Surani et al [54], 2019 | Met | Met | Met | Met | NA | NA | Met | NA | Met | Met | 7/7  (100%) |
| Teh et al [55], 2015 | Met | Met | Met | Met | Met | Met | Met | Not met | Not met | Met | 8/9  (89%) |
| Tolentino and Gephart [56], 2020) | Met | Met | Met | Met | NA | NA | Met | NA | Met | Met | 7/7  (100%) |
| Valk-Draad and Bohnet-Joschko [57], 2022 | Met | Met | Met | Met | NA | NA | Met | NA | Met | Met | 7/7  (100%) |
| Wahyuni et al [58], 2024 | Met | Met | Met | Met | Met | Unclear | Met | Not met | Met | Not met | 7.5/9  (83%) |
| Waneka and Spetz [59], 2010 | Met | Met | Unclear | Met | Unclear | Unclear | Met | Not met | Met | Not met | 6,5/9  (72%) |
| Wong et al [60], 2024 | Met | Met | Met | Met | NA | NA | Met | NA | Met | Not met | 6/7  (86%) |
| Wosny et al [61], 2023 | Met | Met | Met | Met | Met | Met | Met | Not met | Met | Met | 9/9  (100%) |
| Wulff et al [62], 2011 | Met | Met | Met | Met | Met | Not met | Met | Not met | Met | Met | 8/9  (89%) |
| Yang et al [63], 2024 | Met | Met | Met | Met | NA | NA | Met | NA | Met | Met | 9/9  (100%) |
| Young et al [64], 2011 | Met | Met | Met | Met | Not met | Not met | Met | Not met | Met | Not met | 6/9  (67%) |
| Zhang et al [65], 2014 | Met | Met | Met | Met | Met | Met | Met | Not met | Met | Met | 9/9  (100%) |
| Zharima et al [66], 2024 | Met | Met | Met | Met | NA | NA | Met | NA | Met | Not met | 6/7  86% |

Points: Met (1), Unclear (0.5), Not met (0).

*Only applicable for systematic reviews.

References

1. Martin J. Critical appraisal checklist for systematic reviews and research syntheses. Joanna Briggs Institute 2017;7.

2. Abdellatif A, Bouaud J, Lafuente-Lafuente C, Belmin J, Séroussi B. Computerized Decision Support Systems for Nursing Homes: A Scoping Review. Journal of the American Medical Directors Association 2021;22(5):984-994. PMID:33639117

3. Abdolkhani R, Petersen S, Walter R, Zhao L, Butler-Henderson K, Livesay K. The Impact of Digital Health Transformation Driven by COVID-19 on Nursing Practice: Systematic Literature Review. JMIR nursing 2022;5(1):e40348. PMID:35867838

4. Ackerhans S, Huynh T, Kaiser C, Schultz C. Exploring the role of professional identity in the implementation of clinical decision support systems—a narrative review. Implementation Sci 2024;19(1):11.

5. Alobayli F, O’Connor S, Holloway A, Cresswell K. Electronic health record stress and burnout among clinicians in hospital settings: a systematic review. Digital Health 2023;9:20552076231220241.

6. Araujo SM, Sousa P, Dutra I. Clinical Decision Support Systems for Pressure Ulcer Management: Systematic Review. JMIR medical informatics 2020;8(10):e21621. PMID:33064099

7. Arcega J, Autman I, Guzman B, Isidienu L, Olivar J, O’Neal M, Surdilla B. The Human Touch: Is Modern Technology Decreasing the Value of Humanity in Patient Care? Critical care nursing quarterly 2020;43(3):294-302. PMID:32433070

8. Bail K, Gibson D, Acharya P, Blackburn J, Kaak V, Kozlovskaia M, Turner M, Redley B. Using health information technology in residential aged care homes: An integrative review to identify service and quality outcomes. International journal of medical informatics 2022;165:104824. PMID:35792376

9. Borum C. Barriers for Hospital-Based Nurse Practitioners Utilizing Clinical Decision Support Systems: A Systematic Review. Computers, informatics, nursing : CIN 2018;36(4):177-182. PMID:29360699

10. Brown J, Pope N, Bosco AM, Mason J, Morgan A. Issues affecting nurses’ capability to use digital technology at work: An integrative review. Journal of clinical nursing 2020;29(15-16):2801-2819. PMID:32416029

11. Burgess J-M, Honey M. Nurse Leaders Enabling Nurses to Adopt Digital Health: Results of an Integrative Literature Review. Nursing Praxis in Aotearoa New Zealand 2022;38(3). doi:10.36951/001c.40333

12. Cachata D, Costa M, Magalhães T, Gaspar F. The Integration of Information Technology in the Management and Organization of Nursing Care in a Hospital Environment: A Scoping Review. International journal of environmental research and public health 2024;21(8):968.

13. Chua M, Lau XK, Ignacio J. Facilitators and barriers to implementation of telemedicine in nursing homes: A qualitative systematic review and meta‐aggregation. Worldviews on Evidence‐Based Nursing 2024;21(3):318-329.

14. Coffetti E, Paans W, Roodbol PF, Zuidersma J. Individual and Team Factors Influencing the Adoption of Information and Communication Technology by Nurses: A Systematic Review. Computers, informatics, nursing : CIN 2022;41(4):205-214. PMID:36150090

15. Dos Santos FC, Snigurska UA, Keenan GM, Lucero RJ, Modave F. Clinical decision support systems for palliative care management: a scoping review. Journal of pain and symptom management 2023;66(2):e205-e218.

16. Fagerström C, Tuvesson H, Axelsson L, Nilsson L. The role of ICT in nursing practice: an integrative literature review of the Swedish context. Scandinavian journal of caring sciences 2017;31(3):434-448. PMID:27507258

17. Ferdousi R, Arab-Zozani M, Tahamtan I, Rezaei-Hachesu P, Dehghani M. Attitudes of nurses towards clinical information systems: a systematic review and meta-analysis. International nursing review 2021;68(1):59-66. PMID:32608032

18. Fraczkowski D, Matson J, Lopez KD. Nurse workarounds in the electronic health record: An integrative review. Journal of the American Medical Informatics Association : JAMIA 2020;27(7):1149-1165. PMID:32651588

19. Galiano MA, Fergusson MEM, Guerrero WJ, Muñóz MF, Basto GAO, Ramírez JSC, Lozano MG, Sundt AL. Technological innovation for workload allocation in nursing care management: an integrative review. F1000Research 2024;12:104.

20. Glanville D, Hutchinson A, Khaw D. Handheld Computer Devices to Support Clinical Decision-making in Acute Nursing Practice: Systematic Scoping Review. Journal of medical Internet research 2023;25:e39987. PMID:36780222

21. Grechuta K, Shokouh P, Alhussein A, Müller-Wieland D, Meyerhoff J, Gilbert J, Purushotham S, Rolland C. Benefits of Clinical Decision Support Systems for the Management of Noncommunicable Chronic Diseases: Targeted Literature Review. Interact J Med Res 2024;13:e58036. PMID:39602213

22. Huang R, Li H, Suomi R, Li C, Peltoniemi T. Intelligent Physical Robots in Health Care: Systematic Literature Review. Journal of medical Internet research 2023;25:e39786. PMID:36652280

23. Hülsken-Giesler M, Peters M, Müller K. Tracking-Systeme bei Menschen mit Demenz in der stationären Langzeitpflege. [Tracking systems in people with dementia in long-term care - an integrative review]. Pflege 2019;32(6):353-363. PMID:31640470

24. Joseph J, Moore ZEH, Patton D, O’Connor T, Nugent LE. The impact of implementing speech recognition technology on the accuracy and efficiency (time to complete) clinical documentation by nurses: A systematic review. Journal of clinical nursing 2020;29(13-14):2125-2137. PMID:32243006

25. Kangasniemi M, Karki S, Colley N, Voutilainen A. The use of robots and other automated devices in nurses’ work: An integrative review. International journal of nursing practice 2019;25(4):e12739. PMID:31069892

26. Kaye SP. Nurses’ Attitudes Toward Meaningful Use Technologies: An Integrative Review. Computers, informatics, nursing : CIN 2017;35(5):237-247. PMID:27832031

27. Ko M, Wagner L, Spetz J. Nursing Home Implementation of Health Information Technology: Review of the Literature Finds Inadequate Investment in Preparation, Infrastructure, and Training. Inquiry : a journal of medical care organization, provision and financing 2018;55:46958018778902. PMID:29888677

28. Koivunen M, Saranto K. Nursing professionals’ experiences of the facilitators and barriers to the use of telehealth applications: a systematic review of qualitative studies. Scandinavian journal of caring sciences 2018;32(1):24-44. PMID:28771752

29. Konttila J, Siira H, Kyngäs H, Lahtinen M, Elo S, Kääriäinen M, Kaakinen P, Oikarinen A, Yamakawa M, Fukui S, Utsumi M, Higami Y, Higuchi A, Mikkonen K. Healthcare professionals’ competence in digitalisation: A systematic review. Journal of clinical nursing 2019;28(5-6):745-761. PMID:30376199

30. Kosse NM, Brands K, Bauer JM, Hortobagyi T, Lamoth CJC. Sensor technologies aiming at fall prevention in institutionalized old adults: a synthesis of current knowledge. International journal of medical informatics 2013;82(9):743-752. PMID:23845790

31. Kumar S, Merchant S, Reynolds R. Tele-ICU: Efficacy and Cost-Effectiveness Approach of Remotely Managing the Critical Care. The Open Medical Informatics Journal 2013;7:24-29. PMID:24078857

32. Laukka E, Huhtakangas M, Heponiemi T, Kujala S, Kaihlanen A-M, Gluschkoff K, Kanste O. Health Care Professionals’ Experiences of Patient-Professional Communication Over Patient Portals: Systematic Review of Qualitative Studies. Journal of medical Internet research 2020;22(12):e21623. PMID:33289674

33. Lewinski AA, Rushton S, van Voorhees E, Boggan JC, Whited JD, Shoup JP, Tabriz AA, Adam S, Fulton J, Gordon AM, Ear B, Williams JW, Goldstein KM, van Noord MG, Gierisch JM. Implementing remote triage in large health systems: A qualitative evidence synthesis. Research in nursing & health 2021;44(1):138-154. PMID:33319411

34. Li L, Cotton A. A Systematic Review of Nurses’ Perspectives Toward the Telemedicine Intensive Care Unit: A Basis for Supporting Its Future Implementation in China? Telemedicine journal and e-health : the official journal of the American Telemedicine Association 2019;25(5):343-350. PMID:30036155

35. Medina Martin G, Mingo Fernández E de, Jiménez Herrera M. Nurses’ perspectives on ethical aspects of telemedicine. A scoping review. Nursing Ethics 2024;31(6):1120-1139.

36. Matinolli H-M, Mieronkoski R, Salanterä S. Health and medical device development for fundamental care: Scoping review. Journal of clinical nursing 2020;29(11-12):1822-1831. PMID:31512288

37. Mathieson A, Grande G, Luker K. Strategies, facilitators and barriers to implementation of evidence-based practice in community nursing: a systematic mixed-studies review and qualitative synthesis. Primary Health Care Research & Development 2019;20:e6. PMID:30068402

38. Mileski M, Kruse CS, Catalani J, Haderer T. Adopting Telemedicine for the Self-Management of Hypertension: Systematic Review. JMIR medical informatics 2017;5(4):e41. PMID:29066424

39. Mileski M, Brooks M, Topinka JB, Hamilton G, Land C, Mitchell T, Mosley B, McClay R. Alarming and/or Alerting Device Effectiveness in Reducing Falls in Long-Term Care (LTC) Facilities? A Systematic Review. Healthcare (Basel, Switzerland) 2019;7(1). PMID:30934633

40. Morrison K, Hughes T, Doi L. Understanding the use of telehealth in the context of the Family Nurse Partnership and other early years home visiting programmes: A rapid review. Digital Health 2022;8:20552076221123711. PMID:36406154

41. Murali M, Ni M, Karbing DS, Rees SE, Komorowski M, Marshall D, Ramnarayan P, Patel BV. Clinical practice, decision-making, and use of clinical decision support systems in invasive mechanical ventilation: a narrative review. Br J Anaesth 2024;133(1):164-177. PMID:38637268

42. O’Connor S, Yan Y, Thilo FJS, Felzmann H, Dowding D, Lee JJ. Artificial intelligence in nursing and midwifery: A systematic review. Journal of clinical nursing 2023;32(13-14):2951-2968. PMID:35908207

43. Penny RA, Bradford NK, Langbecker D. Registered nurse and midwife experiences of using videoconferencing in practice: A systematic review of qualitative studies. Journal of clinical nursing 2018;27(5-6):e739-e752. PMID:29149507

44. Piscotty R, Kalisch B. Nurses’ use of clinical decision support: a literature review. Computers, informatics, nursing : CIN 2014;32(12):562-568. PMID:25397722

45. Radhakrishnan K, Xie B, Berkley A, Kim M. Barriers and Facilitators for Sustainability of Tele-Homecare Programs: A Systematic Review. Health services research 2016;51(1):48-75. PMID:26119048

46. Saab MM, Landers M, Egan S, Murphy D, Hegarty J. Nurses and Nursing Students’ Attitudes and Beliefs Regarding the Use of Technology in Patient Care: A Mixed-Method Systematic Review. Computers, informatics, nursing : CIN 2021;39(11):704-713. PMID:34238834

47. San TH, Lin SKS, Fai CM. Factors affecting registered nurses’ use of medication administration technology in acute care settings: A systematic review. JBI library of systematic reviews 2012;10(8):471-512. PMID:27820547

48. Setyowati S, Arruum D, Handiyani H, Koestoer RA. Digital Nursing Technology to Achieve Job Satisfaction: A Systematic Review. Open Access Maced J Med Sci 2022;10(F):104-112. doi:10.3889/oamjms.2022.8289

49. Shelley D, Davis D, Bail K, Heland R, Paterson C. Oncology Nurses’ Experiences of Using Health Information Systems in the Delivery of Cancer Care in a Range of Care Settings: A Systematic Integrative Review. In: Seminars in oncology nursing: Elsevier. ISBN:0749-2081. p. 151579.

50. Shiells K, Holmerova I, Steffl M, Stepankova O. Electronic patient records as a tool to facilitate care provision in nursing homes: an integrative review. Informatics for health & social care 2019;44(3):262-277. PMID:30125140

51. Spinewine A, Evrard P, Hughes C. Interventions to optimize medication use in nursing homes: a narrative review. European geriatric medicine 2021;12(3):551-567. PMID:33751478

52. Stevenson JE, Nilsson GC, Petersson GI, Johansson PE. Nurses’ experience of using electronic patient records in everyday practice in acute/inpatient ward settings: A literature review. Health informatics journal 2010;16(1):63-72. PMID:20413414

53. Strudwick G. Predicting nurses’ use of healthcare technology using the technology acceptance model: an integrative review. Computers, informatics, nursing : CIN 2015;33(5):189-98; quiz E1. PMID:25974361

54. Surani Z, John M, Solano López AL, Gbenro V, Slodan L, Strudwick G. Role Played and Strategies Employed by Managers to Support Point-of-Care Nurses’ Use and Adoption of Health Information Technology: A Scoping Review. Nursing leadership (Toronto, Ont.) 2019;32(2):85-101. PMID:31613216

55. Teh RC-A, Mahajan N, Visvanathan R, Wilson A. Clinical effectiveness of and attitudes and beliefs of health professionals towards the use of health technology in falls prevention among older adults. International journal of evidence-based healthcare 2015;13(4):213-223. PMID:26630361

56. Tolentino DA, Gephart SM. State of the Science of Dimensions of Nurses’ User Experience When Using an Electronic Health Record. Computers, informatics, nursing : CIN 2020;39(2):69-77. PMID:32732645

57. Valk-Draad MP, Bohnet-Joschko S. Nursing Home-Sensitive Hospitalizations and the Relevance of Telemedicine: A Scoping Review. International journal of environmental research and public health 2022;19(19). PMID:36232255

58. Wahyuni ED, Nursalam N, Dewi YS, Arifin H, Benjamin LS. Electronic nursing documentation for patient safety, quality of nursing care, and documentation: a systematic review. JPMA. The Journal of the Pakistan Medical Association 2024;74(9):1669-1677.

59. Waneka R, Spetz J. Hospital information technology systems’ impact on nurses and nursing care. The Journal of nursing administration 2010;40(12):509-514. PMID:21084885

60. Wong KLY, Hung L, Wong J, Park J, Alfares H, Zhao Y, Mousavinejad A, Soni A, Zhao H. Adoption of artificial intelligence–enabled robots in long-term care homes by health care providers: scoping review. JMIR aging 2024;7(1):e55257.

61. Wosny M, Strasser LM, Hastings J. Experience of health care professionals using digital tools in the hospital: qualitative systematic review. JMIR human factors 2023;10(1):e50357.

62. Wulff K, Cummings GG, Marck P, Yurtseven O. Medication administration technologies and patient safety: a mixed-method systematic review. Journal of advanced nursing 2011;67(10):2080-2095. PMID:21545642

63. Yang LF, Mu JX, Zhang J, Zang S, Zhang L, Qi JH, Ni CP, Liu Y. Interventions to promote the implementation of pressure injury prevention measures in nursing homes: A scoping review. Journal of clinical nursing 2024;33(5):1709-1723.

64. Young LB, Chan PS, Cram P. Staff acceptance of tele-ICU coverage: a systematic review. Chest 2011;139(2):279-288. PMID:21051386

65. Zhang W, Barriball KL, While AE. Nurses’ attitudes towards medical devices in healthcare delivery: a systematic review. Journal of clinical nursing 2014;23(19-20):2725-2739. PMID:24698308

66. Zharima C, Mhlanga S, Abdulla S, Goudge J, Griffiths F. What engagement strategies are useful in facilitating the implementation of electronic health records in health care settings? A rapid review of qualitative evidence synthesis using the normalization process theory. Digital Health 2024;10:20552076241291286.
